# Extensive Chemometric Investigations of Distinctive Patterns and Levels of Biogenic Amines in Fermented Foods: Human Health Implications

**DOI:** 10.3390/foods9121807

**Published:** 2020-12-05

**Authors:** Martin Grootveld, Benita C. Percival, Jie Zhang

**Affiliations:** 1 Leicester School of Pharmacy, De Montfort University, The Gateway, Leicester LE1 9BH, UK; p11279990@alumni365.dmu.ac.uk; 2 Green Pasture Products, 416 E. Fremont Street, O’Neill, NE 68763, USA; gpplab@greenpasture.org

**Keywords:** biogenic amines (BAs), fermented foods, chemometrics, multivariate (MV) statistical analysis, liquid chromatographic triple quadrupole mass spectrometric (LC-MS/MS) analysis, public health, lipid peroxidation, antioxidants

## Abstract

Although biogenic amines (BAs) present in fermented foods exert important health-promoting and physiological function support roles, their excessive ingestion can give rise to deleterious toxicological effects. Therefore, here we have screened the BA contents and supporting food quality indices of a series of fermented food products using a multianalyte-chemometrics strategy. A liquid chromatographic triple quadrupole mass spectrometric (LC-MS/MS) technique was utilized for the simultaneous multicomponent analysis of 8 different BAs, and titratable acidity, pH, total lipid content, and thiobarbituric acid-reactive substances (TBARS) values were also determined. Rigorous univariate and multivariate (MV) chemometric data analysis strategies were employed to evaluate results acquired. Almost all foods analyzed had individual and total BA contents that were within recommended limits. The chemometrics methods applied were useful for recognizing characteristic patterns of BA analytes and food quality measures between some fermented food classes, and for assessing their inter-relationships and potential metabolic sources. MV analysis of constant sum-normalized BA profile data demonstrated characteristic signatures for cheese (cadaverine only), fermented cod liver oil (2-phenylethylamine, tyramine, and tryptamine), and wine/vinegar products (putrescine, spermidine, and spermine). In conclusion, this LC-MS/MS-linked chemometrics approach was valuable for (1) contrasting and distinguishing BA catabolite signatures between differing fermented foods, and (2) exploring and evaluating the health benefits and/or possible adverse public health risks of such products.

## 1. Introduction

Biogenic amines (BAs) may be biosynthesized and degraded via normal metabolic activities in animals, plants, and micro-organisms. As such, these amines occur in a wide variety of foods, such as fish, meat, and cheese products, and especially in fermented foods such as wines, and yoghurts, etc. [1,2,3]. BA formation in foods usually occurs via the decarboxylation of amino acids [3], of which there are rich sources in these matrices; for example, amino acids are present at very high levels in grapes, and comprise ca. 30–40% of the total nitrogen content of wines [1,2,3].

Metabolic pathways available in lactic acid bacteria, which have the ability to grow and thrive in foods and beverages, generate significant levels of BAs. Routes available for this are the enzymatic production of putrescine from ornithine (catalyzed by ornithine decarboxylase) and/or from arginine via agmatine, a scheme involving prior conversion of the amino acid substrate to agmatine with arginine decarboxylase, followed by transformation of agmatine to N-carbamoylputrescine via the action of agmatine imino-hydroxylase, and then on to putrescine (a second route for its generation involves the conversion of arginine to ornithine and then to this product via the above ornithine decarboxylase-catalyzed route); putrescine to spermine, a process involving the enzyme spermine synthase, and then spermine to spermidine via the actions of spermidine synthase; cadaverine from lysine with lysine carboxylase and a pyridoxal phosphate co-factor; 2-phenylethylamine from phenylalanine catalyzed by aromatic amino acid carboxylases, including tyrosine decarboxylase; tyramine from tyrosine via tyrosine decarboxylase action; histamine from histidine with histidine decarboxylase; tryptamine from tryptophan with trypotophan decarboxylase, another pyridoxal phosphate-dependent enzyme; and trimethylamine from trimethylamine-N-oxide with a trimethylamine-N-oxide reductase (enzymes involved in the conversion of amino acids to BAs are classified as decarboxylase deaminases) [4,5]. BAs may also be biosynthesized from the amination and transamination of aldehydes and ketones [5], and this may be of some relevance to their detection in marine oil products which have been allowed to autoxidize. Indeed, a range of aldehyde species arise from the fragmentation of conjugated hydroperoxydienes, which are lipid oxidation products resulting from the peroxidation of polyunsaturated fatty acids (PUFAs) [6].

Overall, microbial sources of BAs include yeasts, as well as gram-positive and -negative bacteria [7]. The physiological activity of BA synthesis in prokaryotic cells predominantly appears to be associated with bacterial defense mechanisms employed to combat environmental acidity [8,9,10]. Hence, amino acid decarboxylation in this manner enhances survival under harsh acidic stress states [9] via proton consumption, and amine and CO_2_ excretion required to facilitate restorations of internal pH values [11].

As with their biosynthesis, the catabolism of BAs is extensively outlined and reviewed in [5]. In view of their potentially toxic nature, fortunately humans have detoxification enzyme systems which catabolically oxidize BAs in vivo. These enzymes principally comprise monoamine and diamine oxidases (MAOs and DAOs respectively). MAOs are flavoproteins acting by the oxidative deamination of BAs to their corresponding aldehydes, along with hydrogen peroxide (H_2_O_2_) and ammonia. Two different forms of MAO have been identified in humans [5]. DAOs are responsible for histamine catabolism, as is histamine-N-methyltransferase, the latter catalyzing a ring methylation process [5].

Evidence available indicates that BAs may confer a series of human health benefits, which involve their interactions with a wide variety of intracellular macromolecules such as proteins, DNA, and RNA. Indeed, monoamines are typically precursors of neuromodulators and neurotransmitters [12]. Moreover, evidence is accumulating that the polyamines spermine and spermidine are important for sexual function and fertility [13], and polyamines in general are associated with cell growth and differentiation, including protein biosynthesis [14]. Indeed, the generation of BAs in eukaryotic cells is essential, since they are required for the critical biosynthesis of hormones, alkaloids, proteins, and nucleic acids [15]. One further plausible health benefit offered by both monoamine and polyamine forms of BAs is their antioxidant potential [16], and recent studies have shown that they function efficiently in this context, and protect against adverse unsaturated fatty acid peroxidation reactions when present in or supplemented to culinary oils, and other foods rich in PUFAs [6] (details regarding the nature and mechanisms of these antioxidant actions are provided in Section S1 of the Supplementary Materials).

Notwithstanding, the availability of these amines in the diet has not been without its problems. Indeed, adverse toxicological events may be stimulated by the ingestion of foods which are known to provide high concentrations of these agents, and one notable example is the provocation of deleterious hypertensive events in patients receiving therapies with monoamine oxidase inhibitor (MAOI) drug treatments [17]. A further problem is the depression of histamine oxidation, a process which arises from the ingestion of putrescine and agmatine, which serve as potentiators of this process; this promotes histamine toxicity episodes in humans [18]. Moreover, it has been reported that BAs such as putrescine and agmatine give rise to their corresponding carcinogenic nitrosoamines from reactions with nitrite anion, dietary or in vivo [19].

Human sensitivity to BAs is contingent on the availability and activities of detoxifying enzymes featured in BA metabolism, i.e., specific ones such as histamine methyltransferase, and those less specific such as mono- and diamine oxidases. However, since these enzymes are inhibited by different classes of drugs, including neuromuscular blocking agents such as alcuronium, antidepressants [20], and ethanol [21], the accumulation of BAs by the consumption of selected foods and beverages can, at least in principal, give rise to clinical disorders, including the extremely hazardous serotonin syndrome [22]. Further details regarding the adverse health effects associated with the excessive intake of BAs are delineated in Section S2 of the Supplementary Materials.

Current consumer demands for safer and healthier foods has prompted a high level of research investigations focused on BAs, although it should be noted that further studies are required to expand this area. High levels of BAs can build up in fermented foods, including fish, fish sauce, and cheese products. Their biosynthesis and accumulation therein are critically dependent on the availability of bacteria with decarboxylase-deaminase enzyme activities, environmental conditions that are unrestrictive towards their growth and propagation, and the efficient functioning of BA-generating enzymes, together with the presence of sufficient amounts of the relevant amino acid substrates required.

Hence, supporting analytical methodologies for the identification and measurement of BAs are of much importance to the food industry, and also from a public health perspective. Such methods should ideally offer high levels of reliability in order to monitor the potential health benefits offered by fermented food products, and also to circumvent any toxicological risks to consumers arising from their excessive production therein; realistic estimates of their human consumption are also major factors for consideration. To date, BA determinations in foods have represented a major challenge for analytical chemists in view of their non-chromophoric nature, their natural occurrence in complex multicomponent food and biological matrices, and high polarities, factors which are further complicated by a requirement for high analytical sensitivity, potential interferences, and, where relevant, chromatographic separation/resolution issues arising from the presence of many structurally-related agents in samples requiring such analysis [23]. Methods previously available for this purpose, and those for the screening of BA-producing bacteria, are outlined in Section S3 of the Supplementary Materials.

Notwithstanding, in principle, the simultaneous and direct multicomponent determination of BAs by the LC-MS/MS method described here, or a newly-developed strategy focused on largely non-invasive high-resolution proton (^1^H) nuclear magnetic resonance (NMR) analysis [6], serve as valuable assets which, in combination with MV chemometrics strategies, may be employed for the recognition of patterns of these bacterial catabolites which are characteristic of differential bacterial sources of these agents.

Multivariate (MV) data analysis of multicomponent analytical datasets serves as an extremely powerful means of probing and tracking metabolic signatures that are characteristic of differential groups or classifications of samples, and when applied to explore the biochemical basis of human disease etiology, this technique is commonly known as metabolomics [24]. Indeed, to date this combination of multianalyte-MV analysis has been copiously utilized in many biomedical and clinical investigations, mainly for the identification of diagnostic or prognostic monitoring biomarkers for human diseases. However, when applied in a non-biomedical context, the technique can best be described as chemometrics, a technology which also commonly employs many of the MV data analysis strategies used in metabolomics experiments.

In view of the rich sources of BAs in fermented food products, in this study we determined the contents of a total of 8 different BAs in a series of commercially-available fermented fish, fish sauce/paste, vegetable sauce, cheese, wine/vinegar, and cod liver oil (FCLO) products. For this purpose, we employed both univariate and MV chemometrics analysis techniques in order to recognize differential patterns of these catabolites, which may be representative or characteristic of their food, bacterial, metabolic pathway, and/or food processing technology sources. Such analytical information also serves to furnish us with valuable information regarding the provision of these important nutrients in the human diet, and to evaluate the toxicological/adverse health risks presented by the ingestion of fermented foods containing portentously excessive levels of these agents. Currently, a total BA content of *ca*. 1000 ppm is linked to toxicity, and in recommended manufacturing practices, 100 ppm histamine, or a total BA content of 200 ppm, are considered acceptable levels which do not give rise to any associated adverse health effects [25].

These studies were supported by the consideration of further food quality determinations on these fermented food products, which consisted of pH values, titratable acidities (TAs), and total lipid contents, along with an adapted method for determining lipid peroxidation status (thiobarbituric acid-reactive substances (TBARS)).

With the exception of a small number of studies focused on BAs detectable in selected wine products, e.g., [26], to the best of our knowledge this is the first time that MV chemometrics techniques have been applied to explore potentially valuable “between-food classification: distinctions between the concentrations and patterns of BAs in a series of different food products, albeit fermented ones. Therefore, the aims of this investigation are to explore the abilities and reliabilities of LC-MS/MS-based chemometrics analysis techniques to: (1) evaluate the possible public health benefits and/or risks of BAs arising from the human consumption of fermented foods; and (2) effectively compare and distinguish between differing patterns of BA molecules in different classes of fermented food products.

## 2. Materials and Methods

### 2.1. Fermented Food Products

Fermented food products (cheese, fish, fish sauce/paste, vegetable sauce, and wine/vinegar classifications) were randomly selected and purchased from a variety of US retail outlets based in the state of Nebraska. These comprised *n* = 4 fish samples, *n* = 9 fish sauce/paste samples, *n* = 4 vegetable sauce samples, *n* = 5 cheeses, and *n* = 4 wine/vinegar samples (Table 1). Details of the fermentation processes employed by the manufacturers involved were unavailable. Prior to analysis, all samples were stored in a darkened freezer at a temperature of −20 °C for a maximal duration of 72 h.

Fermented cod liver oil (FCLO) was a natural product that was manufactured and kindly donated by Green Pastures LLC, 416 E. Fremont O’Neill, NE 68763, USA for this study. Separate batches (*n* = 10) of this FCLO product were randomly selected by independent visitors to its manufacturing site throughout a 6-month period, as noted in [6].

FCLO products were prepared from the fermentation of Pacific cod livers. Livers were frozen (−20 °C) within 40 min following their harvest from the Pacific Ocean, and then transported to a preparation facility whilst remaining in the frozen state. Fermented CLO was produced from these cod liver sources using a novel and proprietary fermentation technology. Briefly, cod livers were loaded into a fermentation tank, and both salt and the fermentation starter agent were added to induce the process. The tank was completely sealed during the fermentation and, following periods of 28–84 days, the raw FCLO product accumulated and was then isolated from the tank. Following fermentation, products were centrifuged, filtered to remove particulates, and then packed.

On arrival at the laboratory, FCLO product sample batches were de-identified through their transfer to coded but unlabeled universal storage containers. Each sample was subsequently stored in a darkened freezer at −80 °C until ready for analysis (predominantly within 24 h of their arrival).

### 2.2. Analysis of BAs in Fermented Food Product Samples

A liquid chromatographic triple quadrupole mass spectrometric (LC-MS/MS) technique was employed for the simultaneous analysis of up to 11 BAs in fermented food products using an adaption of the LC-MS/MS method reported in [27]. A Shimadzu 8045 LC-MS/MS facility was used for this purpose, the MS/MS detection system for the monitoring and molecular characterization of eluting BA analytes. Primarily, pre-set accurately weighed masses of food samples were shaken with a 20.0 mL volume of 70% (*v*/*v*) methanol/30% (*v*/*v*) water for 20 min, which were then centrifuged at 7000 rpm at 4 °C for another 20 min period. The clear supernatant was subsequently transferred to 1.7 mL volume amber auto-sampler vials for LC-MS/MS analysis. For wine/vinegar and FCLO samples, fixed aliquots were filtered using a 0.45 µm filter paper prior to the above methanol/water extraction stage.

The LC facility comprised a pump, vacuum degasser, auto-sampler, and column compartment, and finally a secondary variable wavelength spectrophotometric detection system was used for these analyses. This system could operate up to 800 bar. The internal standard (IS) utilized was tetra-deuterated histamine (histamine-α,α,β,β−d_4_, (2HCl)), which was purchased from C/D/N Isotopes Inc. (Pointe-Claire, Quebec, Canada). IS *m*/*z* values employed for quantification purposes were 116.1 and 99.0 for precursor and product ions, respectively (112.1 and 95.1 respectively for undeuterated histamine).

A 3-μm 50 × 2.1 mm Pinnacle^®^ DB pentaflurophenyl (PFP) base with propyl spacer column was employed for optimal BA analysis. Mobile phase 1 contained water solutions of the ion-pair reagent trifluoroacetic acid (TFA) (either 0.05 or 0.10% (*w*/*v*)), and mobile phase 2 was acetonitrile containing equivalent TFA concentrations. BA analytes were monitored in positive ion mode for the MS/MS detection system. Reporting limit values for fermented food samples were 1 ppm for all BAs determined.

Authentic BA calibration standards were purchased from Sigma-Aldrich Chemical Co. (St. Louis, MO, USA) (histamine, H7125; cadaverine, 33220; putrescine, D13208; 2-phenylethylamine, P6513; spermidine, 85578; tyramine, T2879; tryptamine, 193747), and Alfa Aesar Inc. (Heysham, UK) (spermine, J63060). BA contents were determined from calibration curves developed with standard solutions of concentrations 0.5, 1.0, 10.0, 50.00, 100.0, 200.0, and 400.0 ppb for each BA.

### 2.3. Total Lipid Analysis

Total lipid (fat) analysis was performed according to the AOAC 922.06 method. Briefly, homogenized samples were treated with HCl, and then washed at least two-fold with both petroleum ether and diethyl ether; solutions arising therefrom were then placed in pre-weighed beaker containers. Subsequently, the lipid-containing ether solutions were evaporated, and the (*w*/*w*) % content of lipid was determined directly from the weight gain of the container.

### 2.4. Determination of Thiobarbituric Acid-Reactive Substances (TBARS) Values

Primarily, accurately-weighed samples were digested with perchloric acid (HClO_4_), and subsequently the resulting clear filtered supernatant solution was reacted with thiobarbituric acid (TBA) for a period of 15–18 h at 27.5 °C according to the method outlined in [28]. The absorbance value at a wavelength of 532 nm was then determined, and TBA-reactive substance (TBARS) values were reported as mg/kg (ppm) units following their quantification from a calibration curve developed with MDA standards.

### 2.5. Titratable Acidity (TA) and pH Value Determinations

Titratable acidity values were determined using the AOAC 947.05 method [29], and pH measurements were made using a modified FO PROC 31 protocol which is based on the USDA PHM method. The latter approach is based on the formation of a homogenized food/water slurry which was allowed to stand prior to pH determination with a probe.

### 2.6. Experimental Design and Statistical Analysis

#### 2.6.1. Univariate Statistical Analysis

The experimental design for univariate analysis of the individual BA, TA, pH, and further variable dataset involved an analysis-of-variance (ANOVA) model, which incorporated 1 prime factor and 2 sources of variation: (1) that “between-fermented food classifications”, a qualitative fixed effect (FF*_i_*); and (2) experimental error (e*_ij_*). The mathematical model for this experimental design is shown in equation 1, in which y*_ij_* represents the (univariate) BA or alternative analyte dependent variable values observed, and μ their overall population mean values in the absence of any significant, influential sources of variation.

y*_ij_* = μ + FF*_i_*+ e*_ij_* (1)

ANOVA was conducted with *XLSTAT2016* and *2020* software. Datasets were autoscaled (i.e., the mean value of each parameter monitored was subtracted from each entry, and the residual then divided by food class standard deviation, which was computed with an (*n* − 1) divisor) prior to analysis. In view of heterogeneities between the intra-sample variances of fermented food classifications, i.e., heteroscedasticities, the robust Welch test was employed to determine statistical significance of differences observed between the mean BA and other food quality variable values for each fermented food group. *post*-*hoc* ANOVA evaluations of the statistical significance of differences between the mean values of individual fermented food groups were performed using the Bonferroni test.

A similar ANOVA-based experimental design was applied to additional design models selected to determine the statistical significance and food class specificities of BA analytes only. For these purposes, the 8 BA dataset, which included those determined in the *n* = 10 batches of the FCLO product, was either constant sum (CS)-normalized or not, and then generalized logarithmically (glog)-transformed, and finally autoscaled prior to analysis. The CS normalization data preparation task was applied in order to evaluate the significance of fermented food classification-dependent BA profile patterns. The non-CS-normalized dataset also included total BA level as a further possible explanatory variable. *MetaboAnalyst* *4.0* (University of Alberta and National Research Council, National Institute for Nanotechnology (NINT), Edmonton, AB, Canada) was utilized for the analysis of these data. Probability values obtained from *post*-*hoc* ANOVA comparisons of individual BA levels between fermented food classes were false discovery rate (FDR)-corrected.

Tests for the heteroscedasticity of ANOVA model residuals (Levene’s test) were performed using *XLSTAT2020* (Addinsoft, Paris, France).

#### 2.6.2. Multivariate Chemometrics and Algorithmic Computational Intelligence (CI) Analyses

Principal component analysis (PCA), partial least squares-discriminatory analysis (PLS-DA), correlation, and agglomerative hierarchical clustering (AHC) analyses of the combined BA dataset were performed using *XLSTAT2016* and *2020* and *MetaboAnalyst* *4.0* [30] software module options. The dataset was generalized glog-transformed, and autoscaled prior *to MetaboAnalyst 4.0* analysis, but only autoscaled for *XLSTAT2016* and *2020* analyses. All these MV analysis strategies were primarily performed on non-CS-normalized data. For the PCA and PLS-DA analyses, limits for significant explanatory variable loadings vectors/coefficients were set at ≤−0.40 or ≥0.40. Validation of PLS-DA models was performed by determining component number-dependent Q^2^ values (predominantly for two classification comparisons), and permutation testing with 2000 permutations. The significance of variable contributions to these models was determined by the computation of variable importance parameter (VIP) values (values >0.90 were considered significant).

Additional PCA analysis was performed in order to explore associations or independencies of individual BAs and other active variables considered, e.g., pH and TA values, total lipid contents, etc. For this purpose, a maximal 5 PC limit was applied, and PCA was then conducted on autoscaled data using varimax rotation and Kaiser normalization. The loadings of each analytical variable on successive orthogonal PCs was then sequentially evaluated. Similarly, this form of PCA was employed to investigate possible inter-relationships and orthogonalities between BA variables analyzed in FCLO batches sampled from the same manufacturing source specified above.

A further PCA model involved its application to the 8 BA dataset alone, which was either CS-normalized or not, glog-transformed, and autoscaled prior to analysis. As noted above, the CS-normalization data preparation step was utilized in order to evaluate the significance of any differential patterns or distributions of BA analytes which may be characteristic of fermented food classifications. This analysis was performed using *MetaboAnalyst* *4.0*.

The random forest (RF) machine-learning algorithm approach was also utilized for classification and discriminatory variable selection purposes (*MetaboAnalyst* *4.0* Random Forest module), with 1000 trees (*ntree*) and 4 predictors selected at each node (mtry) subsequent to tuning. The dataset was randomly split into training and test sets containing approximately two-thirds and one-third of entries respectively. The training set was employed to construct the RFs model, and an out-of-the-bag (OOB) error value was determined to evaluate the classification performance of this. Again, this analysis was performed on the glog-transformed and autoscaled dataset, either with or without prior CS-normalization as specified in the manuscript.

Missing data, specifically total lipid and (TBARS):(total lipid) ratios for 2 × fish sauce/paste, 1 × vegetable sauce, 1 × wine/vinegar, and 1 × cheese samples, were estimated by the support vector machine (SVM) impute technique [31] (*MetaboAnalyst* *4.0*), or supplementation with the explanatory variable column mean values, along with a corresponding reduction in degrees of freedom available for parametric univariate statistical testing (*XLSTAT2016* *or* *2020*).

## 3. Results and Discussion

### 3.1. BA Levels and Food Quality Indices in Fermented Food Products, and Univariate Analysis of These Analytical Data

Mean ± SEM values for the individual and total BA contents of the FF products investigated are provided in Table 2. The major contributors towards the relatively high BA levels observed in fermented cheese samples were cadaverine (mean 60% of total) and tyramine (mean 21.5% of total). Although three of the cheese products analyzed had total BA concentrations of 30–63 ppm, two of them were found to be as high as 666 and 780 ppm, which were markedly above the recommended 200 ppm content limit. The ANOVA Welch test demonstrated that there were highly significant differences between these total BA values (Table 3), as expected (*p* = 2.84 × 10^−4^); such differences were largely explicable by those observed between the cheese and wine/vinegar product classifications investigated.

Hence, characteristic “markers” of fermented cheese samples appeared to be cadaverine and tyramine, which had contents markedly elevated over those of the other fermented food products evaluated, although there were very high intra-fermented food classification variances for these estimates.

**Figure 1:**
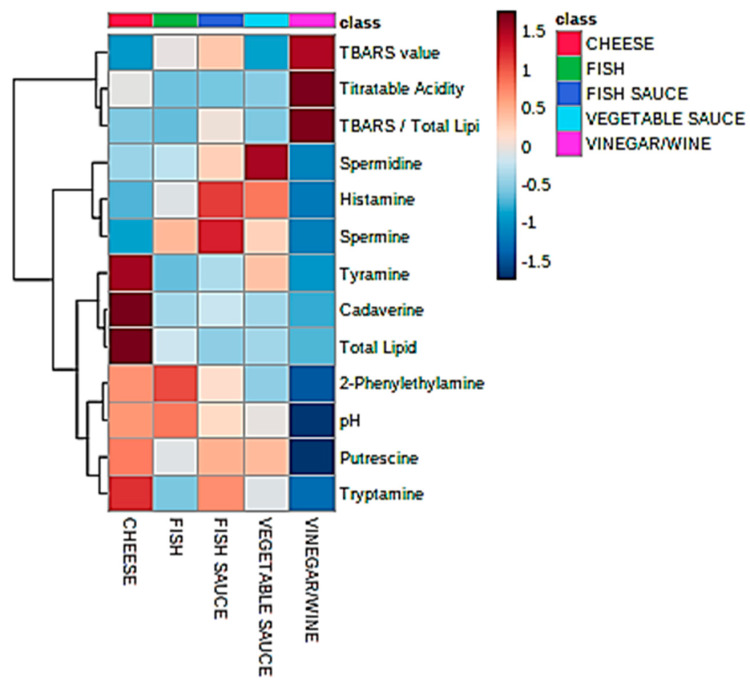
Heatmap diagram displaying the nature, extent and ANOVA-based significance of univariate differences between mean values of all 8 BA and further chemoanalytical food quality variables (near right-hand side *y*-axis) for the fermented cheese (red), fish (green), fish sauce/paste (dark blue), vegetable sauce (pale blue), and wine/vinegar (mauve) products. The complete dataset was glog-transformed and autoscaled prior to analysis, but not CS-normalized. Transformed analyte intensities are shown in the far right-hand side *y*-axis: deep blue and red colorations represent extremes of low and high contents respectively. The left-hand side of the plot shows results arising from an associated agglomerative hierarchical clustering (AHC) analysis of these variables, which reveals two major analyte clusterings, with three sub-clusterings for one of these. The top right-hand side major cluster comprises TBARS level, (TBARS):(total lipid) ratio and TA value, whereas the second contains all other analyte variables, including all BA contents. The first, second, and third sub-clusters within the bottom right-hand side major cluster feature spermine, spermidine, and histamine (the first two of these arising from the same putrescine and metabolically upstream ornithine and agmatine/arginine sources respectively); tyramine, cadaverine, and total lipid; and 2-phenylethylamine, putrescine, tryptamine, and pH respectively.

Univariate statistical analysis performed by ANOVA (robust Welch test derivative), and also *post*-*hoc* Bonferroni test values, demonstrated that the mean values of each food classification examined were significantly or highly significantly different for 7 and 9 of the marker index variables respectively (*p* values ranging from <0.0003 to 0.04 for the former test, Table 3). Figure 1 shows a heatmap of the mean BA contents, and further variables included in this analysis; this clearly displays significantly higher tyramine, cadaverine, putrescine, and tryptamine levels in the fermented cheese products; higher histamine concentrations in the fish sauces/pastes explored, as expected (although vegetable sauces also had quite high levels of this BA); and also greater spermine contents in the fish paste/sauce products (*ca*. 1.5-fold greater than the mean value found for the fish classification, the next highest concentration). The vegetable sauce products had the highest mean spermidine levels, whereas the fermented fish group contained the largest amounts of 2-phenylethylamine detectable.

As expected, mean TA values were significantly greater for the wine/vinegar products than they were for all the other fermented food classes investigated, and correspondingly the mean pH value for the former group was significantly lower than those of all the other fermented foods. Of course, the mean total lipid content of the cheese group (23.3%) was significantly greater than all other food classifications tested (*p* *ca*. 10^−3^), although no significant differences were found for the secondary lipid peroxidation TBARS marker. However, an examination of the mean ratio of TBARS index to total lipid content revealed that this value was markedly greater for the wine/vinegar group than that of all other food product types (Bonferroni-corrected *post*-*hoc* ANOVA tests), and significantly so over that of the cheese samples analyzed, as might be expected in view of the very low fat contents of fermented wine/vinegar samples (for example, it varies from 0.15–0.44% (*w*/*v*) in Zhenjiang aromatic vinegar samples [32]), and potentially substantially inflated TBARS levels resulting from quite high levels of TBA-reactive acetaldehyde and acrolein, amongst other aldehydes, present in such fermented products [33,34,35,36]. Indeed, many other aldehydes are reactive towards the TBA reagent, and also form chromophoric products on reaction with it [28]. Estimates for acetaldehyde in vinegar products can be as high as 1.0 g/kg respectively [33], but such levels are highly variable, with much lower levels being found, e.g., 2.6 mg/L (*ca*. 60 µmol/L) [37].

Acetaldehyde, a volatile flavor component of a variety of foods and beverages such as cheese, yoghurt, and wines [34], represents one of the most abundant carbonyl compounds detectable in wine, and typically accounts for *ca*. 90% of the total aldehydes present; its concentrations therein usually range from 10 to 200 mg/L (predominantly, it is generated as a yeast by-product during alcoholic fermentation processes [35], or from the chemical oxidation of ethanol [36]). However, very high levels of the unsaturated aldehyde acrolein are also present in red wine products [33]. Furthermore, a wide range of further aldehydes have been found to serve as major flavor constituents of traditional Chinese rose vinegar, and these include aliphatic *n*-alkanals such as heptanal, hexanal, nonanal, and dodecanal (ranging from 6–147 µg/kg), with larger amounts of benzaldehyde (851 µg/kg) [38].

Hence, overall these data clearly demonstrated that, in a univariate context, there were indeed significant differences between the mean contents of BAs and further parameters considered for the five classes of fermented food products studied.

**Figure 2:**
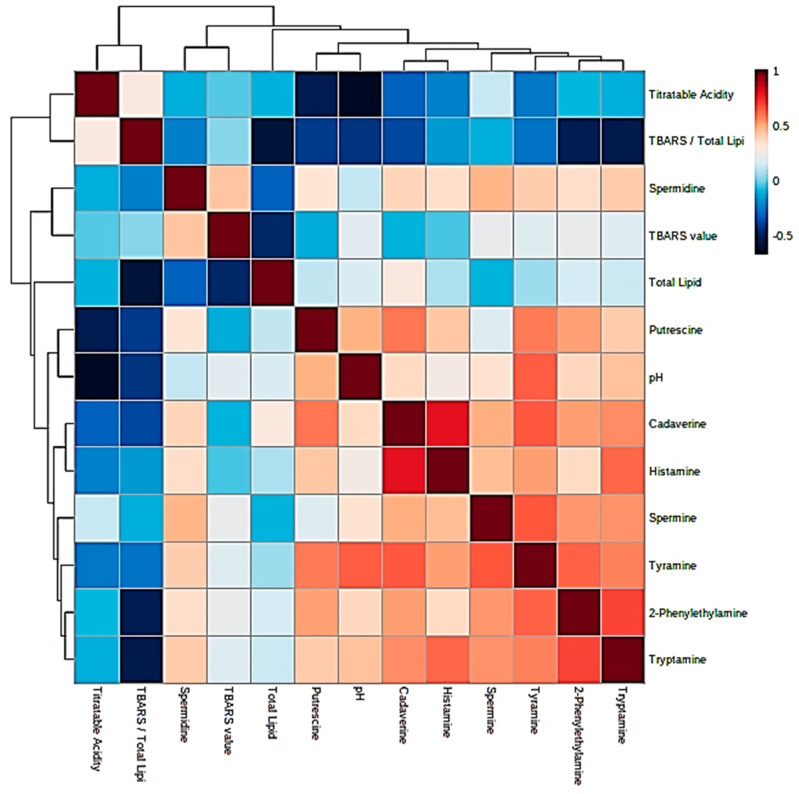
Correlation heatmap displaying positive and negative inter-relationships between BA concentrations, pH and TA values, total lipid contents, TBARS indices and (TBARS):(total lipid) ratios (TBARS/total lipid). The left-hand ordinate and top abscissa axes show AHC analysis based on these Pearson correlations (as a similarity criterion). From the top abscissa axis, of the two major clusterings revealed, that on the right-hand side contains all BA variable levels with the exception of spermidine, together with positively-correlated pH values, whereas the left-hand side one consists of all lipid- and lipid peroxidation-based variables, spermidine concentrations, and TA values.

Prior to the performance of MV statistical analysis of the dataset acquired, simple Pearson correlations were explored between all explanatory variables considered, and Figure 2 shows a correlation heatmap for these relationships. Clearly, there were moderate to strong positive correlations observed between all fermented food BAs present, the strongest observed between 2-phenylethylamine and tyramine (both aromatic BAs), tryptamine and spermine, and most notably, between cadaverine and histamine. Food pH values were found to have the strongest positive correlations with tyramine > putrescine > tryptamine, although spermidine was predominantly uncorrelated with this index. Moreover, as anticipated, TA was strongly negatively correlated with pH value > putrescine > tyramine ≈ histamine contents in that order. TBARS level, however, was largely independent of all BAs and their concentrations, with the exception of spermidine, which exhibited a weak positive relationship with this variable. Similarly, total lipid level was also mainly uncorrelated with all BA contents but was quite strongly anti-correlated with (TBARS):(total lipid) ratio and non-lipid-normalized TBARS value (both expected). The (TBARS):(total lipid) ratio was either strongly or moderately anti-correlated with all BA levels, and this may provide an indication of their potential antioxidant functions. In view of the complexity of these inter-relationships, the MV PCA and PLS-DA techniques were employed to explore them further.

### 3.2. Principal Component Analysis (PCA) of the Multivariate Fermented Food Dataset

**Figure 3:**
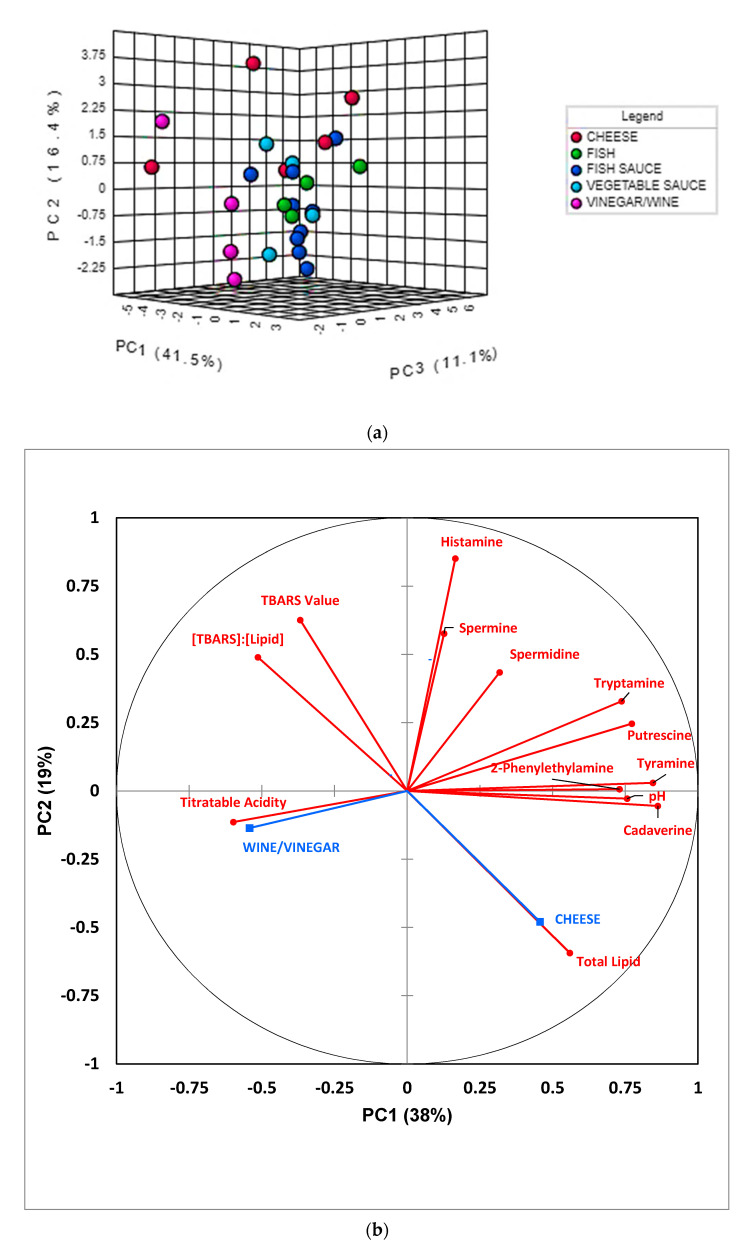
(**a**) 3D PCA scores plot of PC3 vs. PC2 vs. PC1, showing some degrees of distinction between different fermented food classes, i.e., those of cheese, fish, fish sauce/pastes, vegetable pastes, and wines/vinegars (particularly that between the wine/vinegar classification and all others). (**b**) Preliminary correlation circle diagram displaying correlations between all explanatory variables considered, and PCs 1 and 2 in a PCA model applied to the complete autoscaled (standardized) dataset. Active variables are depicted in red, whereas two of the supplementary variable classifications (cheese and wine/vinegar) are shown in blue. Variance contributions for PC1 and PC2 are indicated.

PCA was primarily conducted in order to acquire an overview of the degree of distinctiveness between, i.e., clustering of, the fermented food classifications investigated, and also to identify any potential data outliers. An examination of two-dimensional (2D) scores plots from this analysis demonstrated that no significant outliers were detectable, and that PCs 1, 2, and 3 accounted for 41.5, 16.4, and 11.1% of the total variance respectively for the complete dataset which was glog-transformed and autoscaled. 2D and three-dimensional (3D) scores plots featuring these two most important PCs revealed that there was a reasonable level of distinction between the wine/vinegar and all other food product groups, and also between the cheese and fish classifications (Figure 3a); however, distinctions between the fish, fish sauce/paste, and vegetable sauce groups were not found, there being a significant degree of overlap between them. Notwithstanding, the sample sizes of the fermented fish and vegetable sauce groups involved were quite limited. A corresponding preliminary correlation circle diagram is shown in Figure 3b. Clear observations from this diagram are that (1) 2-phenylethylamine, tyramine, and cadaverine, and to a lesser extent, putrescine and tryptamine, are all correlated with PC1, and this observation indicates their communality in this model; (2) food pH values are also strongly correlated to PC1, and this indicates that higher values of this parameter may arise from the basicity of the above BAs (gas-phase primary amine basicity values increase with the length of its carbon chain substituents in view of their electron-donating positive charge-stabilizing effects—such values also increase with progression from primary to secondary to tertiary alkylamines [39]); (3) an at least partial correlation of histamine contents with PC2, which indicates distinction of this BA from those aligned with PC1; (4) an inverse correlation (anti-correlation) of total lipid level with the (TBARS):(total lipid) ratio index, as might be expected; and (5) a strong anti-correlation of TA value with BA levels, particularly tryptamine and putrescine, and this suggests that these amines serve to offer neutralization potential against acidic fermented food products. Also notable from this Figure are very strong correlations between the fermented food supplementary variable cheese and total lipid content, and between wine/vinegar and TA value, as indeed expected.

A more detailed analysis of these PCA loadings was made with the application of varimax rotation, Kaiser normalization, and a maximal number of 5 PCs considered. For this model, such variable loadings, and the percentage of total variance accounted for by each PC are available in Table 4. This analysis revealed that cadaverine, tryptamine, 2-phenylethylamine, and tyramine all strongly and positively loaded on PC1, spermidine and histamine strongly and positively loaded on PC3 (along with a more minor contribution from 2-phenylethylamine), and putrescine and spermidine loaded strongly and positively on PC5, albeit also with histamine to a much lesser extent. Interestingly, all aromatic BAs strongly loaded on PC1, as observed above (Figure 3b), whereas spermidine and its metabolic precursor putrescine both co-loaded onto the same PC (PC5).

The TBARS secondary lipid oxidation index, along with its value normalized to total food lipid content, both loaded strongly and positively on PC2, as might be expected, although histamine also contributed somewhat towards this PC. Moreover, TA and pH values powerfully loaded on PC4 negatively and positively respectively, as would be expected from their anticipated negative correlation in fermented food products (putrescine also made a moderate positive contribution towards this component). Total lipid content was found to load significantly on PCs 1 and 5, positively and negatively so, respectively.

In a related study focused on PCA of both BAs and polyphenolics in Hungarian wines, Cosmos et al. [26] found that PC scores successfully clustered differential groups of these product classes, and that PC loadings vectors displayed significant patterns of BA and polyphenol levels. However, it should be noted that for this analysis, spermidine, and tyramine strongly loaded on PC1 (positively and negatively, respectively), agmatine and the sum total BA concentration loaded strongly and positively on PC2, spermine and cadaverine both strongly and negatively loaded on PC3, and that histamine loaded strongly and positively on PC4 alone. These associations between the BA analytes tested did not correspond to those found in the present study, although in the above MV analyses we elected not to include the total summed BA concentration value. Furthermore, our study also included the determinations of 2-phenylethylamine and putrescine, and not agmatine, but that reported in [26] monitored the latter BA but not 2-phenylethylamine and putrescine. However, as noted by the authors of [26], these PC loadings are only applicable to one region of Hungarian wine production, and their results will not be readily transferable to others, let alone other classes of fermented foods, especially in consideration of the often highly variable methods of fermentation, sources of fermentative micro-organisms, and conditions employed for these purposes. Notwithstanding, these researchers also concluded that in view of the loading patterns of BAs observed, it was unnecessary to measure all BA variables for quality assessments, and that only one per orthogonal PC was sufficient to provide acceptable levels of distinction between different sub-classes of such wines.

From this analysis, the unambiguously strong loadings vectors of the aromatic BAs 2-phenylethylamine and tyramine on PC1 provide evidence that they may indeed arise from the same biological and/or metabolic sources; however, this observation may also be rationalized by the natural production of tyrosine from phenylalanine, i.e., that involving the possible hydroxylation of the latter substrate to the former catalyzed by the enzyme phenylalanine hydroxylase (PAH) potentially available in fermentative lactobacilli employed for the production of fermented food products, followed by enzymatic transformation of the tyrosine product to tyramine by fermentative bacteria. To date, PAH is the only known aromatic amino acid hydroxylase found in bacteria [40].

The loadings of spermine and spermidine on different orthogonal PCs (PC3 and PC5, respectively) is not simply explicable, although the co-loading of spermidine’s metabolic precursor putrescine on PC5 is consistent with them being featured in the same metabolic pathway. However, the co-loadings of BAs on differential PCs, particularly PC1, may reflect their engenderment from identical or related bacterial sources.

Notably, PC2 was dominated by powerful loading contributions from TBARS level and (TBARS):(total lipid) ratio (both positive), and PC4 by strong loadings from TA and pH values (negative and positive loadings vectors, respectively). These inter-relationships are, of course, expected, and are consistent with the data presented in Figure 3b. PC5 was retained in the model since it was the only one available which had a strong loading contribution from spermidine.

### 3.3. Distinction of Fermented Food Classifications Using PLS-DA

**Figure 4:**
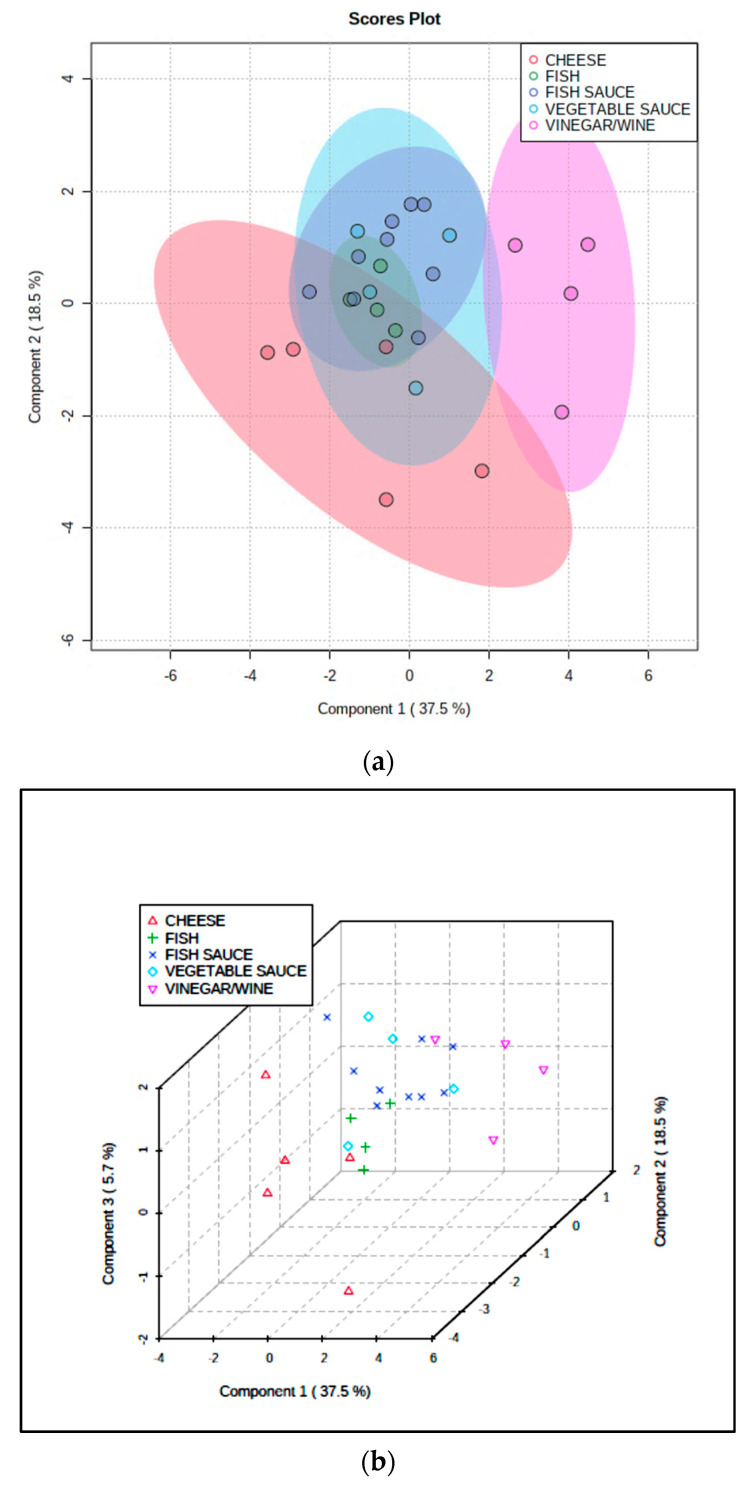
(**a**,**b**). 2D and 3D PLS-DA scores plots (PC2 vs. PC1, and PC3 vs. PC2 vs. PC1, respectively) revealing strong distinctions between the cheese, wine/vinegar, and a considered combination of fish, fish sauce/paste and vegetable sauce fermented food groups ((**a**) also shows 95% confidence ellipses for each fermented food classification). Little or no distinction between the latter three fermented food groups were discernable using this MV analysis approach.

Similarly, PLS-DA of the dataset revealed an effective discrimination between the cheese and wine/vinegar classifications, although the fish, fish sauce/paste and vegetable sauce sample PC score datapoints were again unresolved; however, a visualized combination of these three fermented food classifications was at least partially resolved from the fermented cheese group (Figure 4). Permutation testing of the PLS-DA model confirmed its ability to distinguish between all the differing fermented food classifications evaluated (*p* = 0.022). For this model, key discriminatory variables were selected on the basis of their variable importance parameters (VIPs), and these were total lipid content (1.81) > cadaverine content (1.61) > (TBARS):(total lipid) ratio (1.36) > TA value (1.24) > histamine content (1.14) > 2-phenylethylamine content (0.78); data were glog-transformed and autoscaled prior to analysis. The top three discriminators largely arise from differential levels of lipids, cadaverine, and (TBARS):(total lipid) ratio between each of the fermented food groups, e.g., for the total lipids and cadaverine variables, the cheese content was significantly greater than that of all other fermented food groups, and for the above ratio, its value was significantly greater in the wine/vinegar group than it was in all other groups.

The quite strong distinctions observed between the cheese, wine/vinegar, and fish-fish sauce/paste-vegetable sauce composite products is readily explicable by significant or even substantial differences between the higher contents of cadaverine, tyramine, and, to a lesser extent, tryptamine in cheese, than those of the four other fermented food product classes. Further key discriminators are TA, pH, and total lipid contents, the latter of which is, of course, much higher in the cheese group.

### 3.4. RF Modelling of Fermented Food Classifications

Application of the RF CI classification technique was found to be only partially successful for the classification of the different fermented food groups investigated. Using the models described in Section 2.6.2, this approach correctly classified 4/4 wine vinegar, 6/9 fish sauce/paste, and 3/5 cheeses, but 0/4 for both fish and vegetable sauce products.

### 3.5. PCA of FCLO BAs

The FCLO product considered was primarily investigated separately since only BA contents, and not parameters such as pH and TA were available for it. Moreover, its total lipid content is, of course, not far removed from a value of 100%, and therefore it would be inappropriate to test this index in the above MV analysis models (similarly, total lipid level-normalized TBARS values would also be inappropriate to test in these systems). However, it was possible to explore inter-relationships between FCLO BA concentrations and/or their orthogonality status using a rigorous PCA approach featuring varimax rotation and Kaiser normaliszation in order to maximize success with the assignment of individual BA variables to PCs.

Table 5 lists the BA contents of *n* = 10 FCLO product batches. The total concentrations of BAs in these samples was higher than the recommended ”limit” of 200 ppm in only two out of ten batches of the samples tested, albeit marginally so (only 14 and 20% higher). Similarly, bioactive histamine was completely undetectable in this product. As noted in [6], all BAs monitored were completely undetectable in three other natural, albeit unfermented, CLO products included for comparative purposes. All BAs tested were found to be reasonably soluble in FCLO lipidic matrices, and also in 1/3 (*v*/*v*) diluted solutions of this product in deuterochloroform (C^2^HCl_3_), presumably as the uncharged species with their amine functions deprotonated (solubility in these media is expected to increase with increasing amine function substituent chain length and hydrophobicity).

PCA performed on the FCLO BA dataset revealed that cadaverine, putrescine, and tryptamine all loaded strongly and positively on the first of two automatically-selected PCs (PC1), whereas the aromatic BAs 2-phenylethylamine and tyramine loaded strongly and positively on the second (PC2), along with spermidine (Table 6). These data displayed some consistency with PC loading values obtained on the full fermented food dataset (Table 4), which had 2-phenylethylamine and tyramine both strongly loading on one PC (PC1). However, such levels will, of course, be critically dependent on the microbial fermentation sources, parameters employed for fermented food production, and production conditions for these processes.

### 3.6. MV Chemometric Analysis of BA Data Only: Recognition of Fermented Food Class-Distinctive BA Patterns Using CS-Normalization

Additionally, we conducted univariate and MV analyses of datasets which were restricted to the BA profiles only, but also included the *n* = 10 FCLO samples reported above. Additionally, these analyses were performed with and without application of constant sum (CS) normalization. The CS-normalized data format was employed in order to facilitate the recognition of fermented food class-specific BA patterns. For the non-CS-normalized format, the total BA content value was also included as an explanatory variable, as indeed it was in [26].

**Figure 5:**
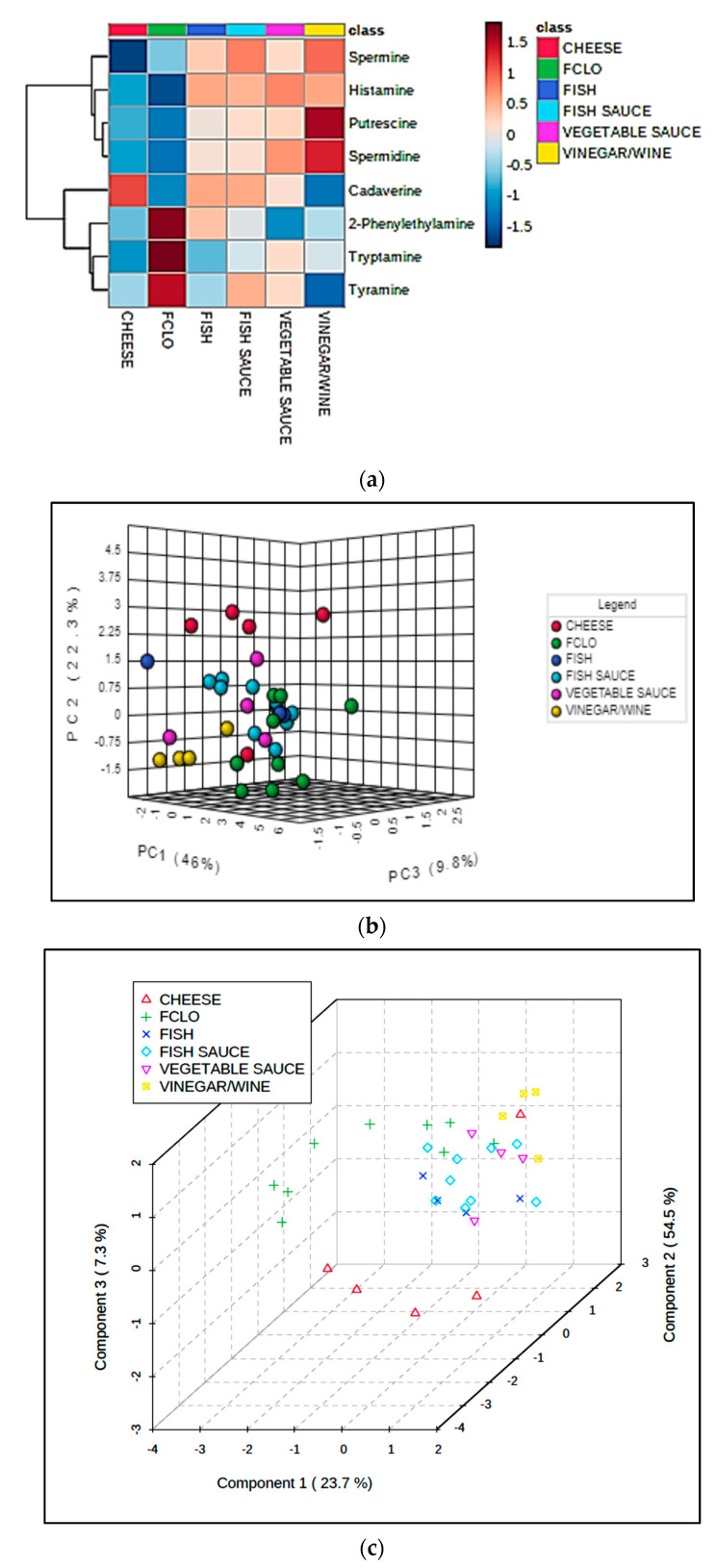
(**a**) Heatmap diagram displaying the most univariately-significant differences between mean values of eight BA explanatory variables (near right-hand side *y*-axis) for the fermented cheese (red), FCLO (green), fish (dark blue), fish sauce/paste (pale blue), vegetable sauce (purple), and wine/vinegar (yellow) products. The complete BA dataset was CS-normalized, glog-transformed, and autoscaled prior to analysis. AHC analysis shown on the left-hand side ordinate axis demonstrated two major analyte clusterings, the upper one consisting of putrescine, spermidine, and spermine pathway biomolecules (and histamine), whereas the lower one features all aromatic BAs, along with cadaverine. (**b**) 3D PCA PC3 vs. PC2 vs. PC1 scores plot for the same CS-normalized dataset shown in (**a**), showing reasonable or strong distinctions between the cheese, wine/vinegar and FCLO fermented food classes. (**c**) 3D PLS-DA PC3 vs. PC2 vs. PC1 scores plot for the corresponding non-CS-normalized dataset, which also incorporated total BA content as a potential explanatory variable (again, effective distinctions between the cheese, FCLO, and wine/vinegar classes were notable).

Firstly, ANOVA performed on the CS-normalized, glog-transformed, and autoscaled dataset found very highly significant, albeit FDR-corrected *p* values for three of the sum-proportionate mean BA concentration differences observed between the fermented food classifications explored in this manner. Notably, these differences were observed for cadaverine, 2-phenylethylamine, and tryptamine (Table 7), and *post*-*hoc* testing revealed that for cadaverine, the cheese products had significantly greater proportionate levels than three others, and for both 2-phenylethylamine and tryptamine, FCLO had significantly higher ones than all other products examined. These differences in CS-normalized values are readily visualizable in the form of an ANOVA-based heatmap (Figure 5a), which revealed characteristic BA signatures for three of the fermented food product classifications. Clearly, the cheese, FCLO, and wine/vinegar sampling groups have high proportionate levels of cadaverine, 2-phenylethylamine/tyramine/tryptamine (all aromatic BAs), and metabolic pathway-associated putrescine/spermidine/spermine, respectively. However, when evaluated in this univariate system, ”between-fermented food class” mean differences observed for putrescine, spermine, spermidine, histamine, and tyramine were not found to be statistically significant.

Secondly, both PCA and PLS-DA models were employed, and these approaches were successful in providing evidence for the MV distinctiveness of the FCLO, cheese, and wine/vinegar groups; however, as noted for the analyses conducted on the combined BA/further food quality parameter dataset, unfortunately no distinctions were observed between the fermented fish, fish sauce/paste, and vegetable sauce products (Figure 5b,c).

For the CS-normalized dataset (without total BA concentrations as an additional variable), PLS-DA variable importance parameter (VIP) values were in the order spermidine (1.48) > putrescine (1.34) > spermine (1.20) > histamine (1.06) > 2-phenylethylamine (0.94), whereas those for the non-CS-normalized dataset were spermidine (1.56) > spermine (1.35) > 2-phenylethylamine (1.32) > putrescine (0.84) (total BA level was a very poor predictor variable for the latter). As expected, there were significant differences between the sequential orders of these values when prior CS-normalization was implemented.

Moreover, for the PLS-DA model adopted without CS-normalization, histamine, spermidine, and spermine contents all loaded significantly on component 1 (loading vector coefficients 0.48, 0.57, and 0.47 respectively); 2-phenylethylamine, cadaverine, tyramine, and total BA levels on component 2 (loadings vector coefficients 0.42, −0.61, −0.57, and −0.61 respectively); 2-phenylethylamine and tryptamine levels on PC3 (loadings vector coefficients 0.50 and 0.57 respectively); and putrescine and spermine on PC4 (loadings vector coefficients 0.75 and −0.73 respectively). For this dataset, a four-component model was found to be most effective (permutation *p* value 0.0055)

Importantly, it should be noted that one now common issue in chemometrics/metabolomics experiments is the occurrence of a univariately-insignificant variable which remains multivariately- significant. Such observations are readily rationalized, firstly by the complementation (i.e., correlation) between explanatory variables, i.e., separately they do not, but when combined together as a MV composite (e.g., as a sufficiently-loading PC variable), they do serve to explain “between-classification” differences detected; secondly, consistency effects arising from the “masking” of potential univariately-significant differences by high levels of biological source sampling and/or measurement variation may be responsible (such variation may be averaged out via the conversion of datapoints to orthogonal component scores as in the PCA and PLS-DA models applied here); and thirdly, relatively small sample sizes for each classification involved (fermented foods in this case)—unfortunately, strategies applied to correct for FDRs promote the risk of statistical type II errors (i.e., false negatives) [24].

The PLS-DA evaluation was then extended and performed for pairwise comparisons of the differing fermented food classifications (CS-normalized dataset only). Firstly, as expected, Q^2^ values for the fish vs. fish sauce/paste, fish sauce/paste vs. vegetable sauce, and fish vs. vegetable sauce comparisons were all moderately negative, and *p* values for associated permutation tests were all >0.10. However, these values for the wine/vinegar vs. FCLO, and FCLO vs. cheese two classification model comparisons revealed that Q^2^ (permutation *p* values) indices for these comparisons were 0.71 (0.059) and 0.72 (0.090), but only 0.38 (0.16) for the wine/vinegar vs. cheese one (values were based on models containing two, five, and one components respectively). Hence, these results provide some evidence for the success of this strategy in distinguishing between the FCLO product, and both the cheese and wine/vinegar ones, although permutation test *p* values obtained for these models were a little higher than the 0.05 significance level, i.e., they were close to statistical significance.

**Figure 6:**
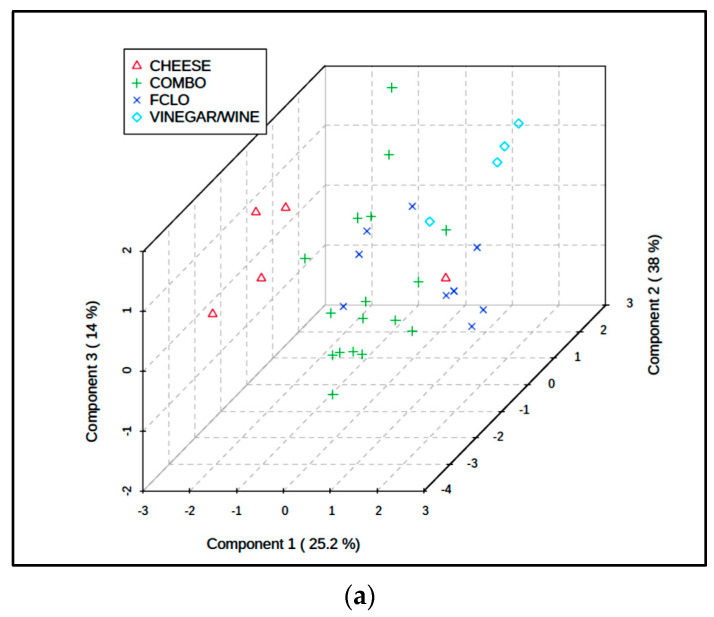

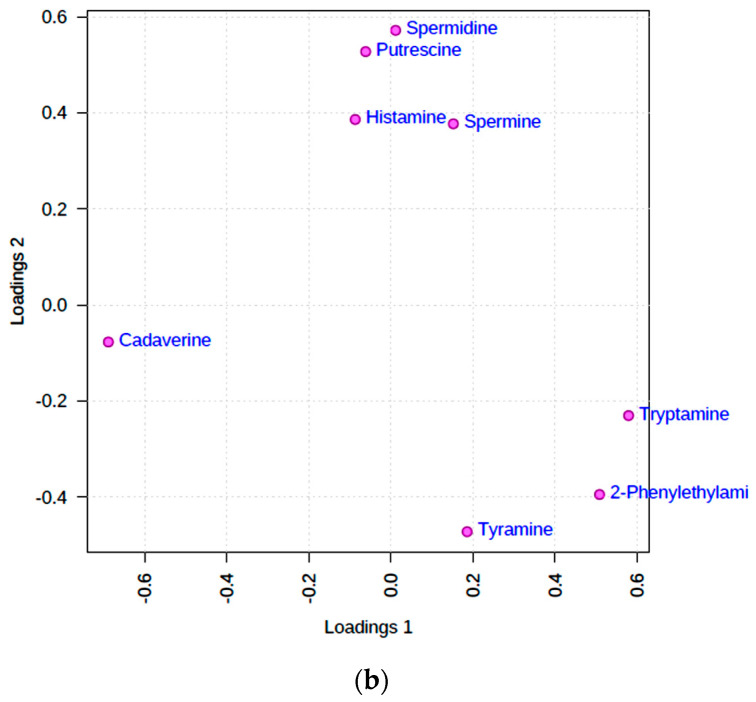
PLS-DA evaluation of revised dataset with combined fish, fish sauce/paste, and vegetable sauce classifications (abbreviated COMBO); CS-normalization was applied to the dataset prior to analysis. (**a**) 3D PLS-DA component 3 vs. component 2 vs. component 1 scores plot revealing some clustering of the fermented food classifications (i.e., cheese, wine/vinegar, FCLO, and COMBO). (**b**) Corresponding component 2 vs. component 1 loadings plot for this PLS-DA analysis.

We then elected to statistically combine the fish, fish sauce/paste, and vegetable sauce groups, and repeated the PLS-DA modelling in order to compare the sauce/fish composite, cheese, FCLO, and wine/vinegar groups using the CS-normalized dataset. This analysis exhibited a quite high level of classification success (Figure 6a); Q^2^ for this comparative four-classification analysis was 0.44, and a PLS-DA permutation test confirmed its significance (*p* = 0.031). The loadings of each BA variable on PLS-DA components 1 and 2 is shown in Figure 6b, and this demonstrates three groups of these predictors: the first with highly positive component 1 and highly negative component 2 loadings (all aromatic BAs, i.e., 2-phenylethylamine, tyramine, and tryptamine); the second with low to intermediate positive component 1 but highly positive component 2 loadings (metabolically-related putrescine, spermidine, and spermine, together with histamine); and the third with highly negative loadings on component 1, but negligible loadings on component 2 (cadaverine only). These grouped BA loadings vectors were very consistent with other observations made from the MV analysis of these data as a full six fermented food classification dataset. Specifically, they are completely reflective of the patterns of BA “markers” found in fermented FCLO, wine/vinegar, and cheese products respectively (Figure 5a).

Finally, RF analysis of this revised dataset showed that this approach had an at least reasonable level of classification success, with all (10/10) FCLO and 88% (15/17) of the fish/sauce combination samples being correctly classified; notwithstanding, only 60 and 50% of the cheese and wine/vinegar fermented food products, respectively, were.

### 3.7. Scientific Significance and Human Health Implications of Results Acquired

Results acquired from the combined applications of univariate and MV chemometrics techniques in this study clearly demonstrated that the latter strategy was valuable for distinguishing between fermented wine/vinegar products and cheeses, and the discrimination between both of these food classes from either fish, fish sauce/paste, or vegetable sauce products (or a statistical combination of them) was possible on the basis of their BA, total lipid, pH, and TA values; nevertheless, such techniques were not readily able to distinguish between the latter three fermented food classes. However, a rigorously-constrained univariate analysis method selected to overcome complications arising from intra-food classification heteroscedasticities and FDRs was able to successfully distinguish between the vegetable sauce and fish groups through significantly higher and lower levels of spermidine and 2-phenylethylamine, respectively, present in the former class. Moreover, experimental results indicated that cadaverine, tyramine, putrescine, and tryptamine concentrations may all contribute significantly towards food pH values in view of their strong positive correlations with this parameter found, together with corresponding negative ones with TA values (Figure 3b).

Moreover, BA-targeted univariate and multivariate analyses of CS-normalized data was found to be valuable for providing useful discriminatory information, which highlighted the characteristic patterns of BA biomolecules, which may be valuable for further investigations of the particular nature and/or geographic origins of fermented foods, and the mechanisms involved in their formation. Indeed, the present study found that such patterns comprised cadaverine only for cheese samples, three aromatic BAs (2-phenylethylamine, tyramine, and tryptamine), for FCLOs (sourced from fermented cod livers), and those from the sequential metabolic pathway which transforms the amino acid substrates ornithine or arginine to spermine (i.e., putrescine, spermidine, and spermine itself) for wine/vinegar products. Such idiosyncratic, fermented food product-dependent signatures for CS-normalized fermented food BA concentrations may serve to provide valuable information regarding the fermentative bacterial sources, routes involved in fermentation, and product manufacturing conditions employed for them.

For the putrescine → spermidine → spermine metabolic pathway, which was identified as representing a wine/vinegar-specific one from analysis of the CS-normalized dataset, and which accounted for >70% of total BAs in this fermented food class (Table 2), both positive or negative correlations could arise between a BA catabolite and its immediate upstream precursor, but not necessarily between the terminal spermine metabolite and that upstream of its spermidine substrate (i.e., putrescine).

With regard to toxic concentrations and health risk recommendations available in [25], it should be noted that all mean histamine levels determined in the fermented food samples tested here lie markedly below the recommended 100 ppm limit for it (with no single product exceeding this value—the highest level observed was 57 ppm in one of the fish sauce products assessed). Furthermore, with the exception of the cheese products evaluated, the mean total BA values all food groups were <200 ppm, the wine/vinegar classification substantially so (Table 2). However, although three of the cheese products tested had total BA contents of <200 ppm, two of them had levels ranging from 600–800 ppm, and therefore their dietary consumption may present a health risk for susceptible individuals.

Mean BA concentrations for the FCLO product examined ranged from 0 (histamine) to only 34 ppm (2-phenylethylamine), with the highest levels observed for the most predominant species, 2-phenylethylamine and tyramine, being 103 and 88 ppm. Since the United States of America’s recommended dietary intake of health-friendly, highly unsaturated omega-3 (O-3) fatty acids (FAs) is a maximum of 1.0 g/day [41], and the oil explored here contains a mean of 29% (*w*/*w*) total O-3 FAs (predominantly the sum of eicosapentaenoic and docosahexaenoic acids) [6], then daily consumption of 100/29% × 1.0 g = 3.45 g of this FCLO product would provide estimated absolute maximal daily intake levels of 3.45 × 103 µg = 355 µg, and 3.45 × 88 µg = 304 µg of 2-phenylethylamine and tyramine, respectively. Based on the 10 samples of this product analyzed, estimated mean daily intakes of these BAs will be 111 and 95 µg only. Therefore, it appears that daily consumption of this product at the recommended U.S.-recommended dosage levels will certainly not provide any health risks to consumers, even if they are susceptible to the adverse effects experienced by their excessive intake (e.g., migraines induced by 2-phenylethylamine).

As noted above, one potentially important health benefit offered by the ingestion of dietary BAs is their novel antioxidant properties, both for the prevention of food spoilage during storage or transport episodes, but also in vivo following their ingestion. Indeed, our laboratory recently explored the powerful antioxidant capacities of BA-containing natural FCLO products, and their resistivities to thermally-mediated oxidative damage to unsaturated FAs therein, particularly O-3 PUFAs [6]. These marine oil products, which arise from the pre-fermentation of cod livers (Section 2), were indeed found to display a very high level of antioxidant activity, and PUFAs therein were also more resistant towards thermally-mediated peroxidation than other natural cod liver oil products evaluated. Resonances assignable to aromatic BAs, specifically those arising from 2-phenylethylamine and tyramine, were directly observable in the ^1^H NMR profiles of ca. 1/3 (*v*/*v*) diluted solutions of these products with C^2^HCl_3_. Additionally, corresponding spectra acquired on both ^2^H_2_O and C^2^H_3_O^2^H extracts of these oils confirmed the presence of both these BAs, together with a series of others, both aromatic and aliphatic. In the present study, mean concentrations of 2-phenylethylamine and tyramine detectable in these products were found to be 34 and 24 ppm, respectively. Antioxidant actions of the phenolic BA antioxidant tyramine found in this FCLO product may be explicable by its chain-breaking antioxidant effects, and this may offer contributions towards the potent resistance of PUFAs, particularly O-3 FAs, present therein. However, in view of the absence of a phenolic function in 2-phenylethylamine, its antioxidant potential is likely to involve an alternative radical-scavenging mechanism, presumably that involving O_2_-consuming carbon-centered pentadienyl radical species, as found in [22,23].

The TBARS method employed here to determine the lipid peroxidation status of fermented foods, which involved an extended low temperature equilibration process [28], successfully avoids the artefactual generation of TBA-reactive aldehydes, including malondialdehyde (MDA), during commonly-employed alternative protocols for this assay system, which generally involve a short (ca. 10–15 min) heating stage at 95–98 °C in order to develop the monitored pink/red chromophore rapidly. However, from an analysis of TBARS and (TBARS):(total lipid) ratios determined on the preliminary FCLO-excluded fermented food samples, there appears to be only little evidence for the ability of BAs to offer any protection against lipid peroxidation in such products. Although Table 3 shows that the above ratio is significantly greater in the wine/vinegar group, this observation is perceived to be derived from their very low lipid contents, and the presence of a range of non-MDA TBA-reactive aldehydes present therein, including acetaldehyde and acrolein, for example, although these are also lipid oxidation products. Moreover, despite taking steps to avoid the artefact-generating heating stage of this assay, this test still remains poorly specific in view of the reactions of a variety of non-aldehydic substrates to react with it to form interfering chromophores, which also absorb at a monitoring wavelength of 532 nm. Nevertheless, TBARS level appeared to be positively correlated with fermented food spermidine concentration (Figure 2), and both this lipid peroxidation index and its lipid-normalized value appeared to be positively correlated with fermented food histamine content (loading on PC2, Table 4). However, in view of the many complications associated with this TBARS lipid peroxidation index, which offers only a very limited and still often erroneous viewpoint on the highly complex lipid peroxidation process [42], such observations cannot be rationally considered at this stage. As expected, the lipid-normalized TBARS value was negatively correlated with total lipid content (Figure 3b). The latter variable also appeared to be negatively correlated with histamine and putrescine levels (loading on PC5, Table 4). Unfortunately, results from unspecific TBARS assays are still widely employed as important quality indices throughout the food industry.

One quite surprising observation made in the current study was the detection of lipids, albeit at low levels, in wine and vinegar samples. Notwithstanding, as noted above, FAs have been detected in Zhenjiang aromatic vinegar products at similar contents to those found here [32]. Furthermore, Yunoki et al. [43] explored the FA constituents of some commercially-available red wine products, and found that lipid constituent concentrations varied from 27 to 96 mg/100 mL for *n* = 6 domestic (Japanese) wines, and 31 to 56 mg/100 mL for *n* = 6 foreign products, and that a total of 12 different FAs were detectable, mainly saturated ones. Although the extraction method described in the latter report was a 2:1 chloroform:methanol (Folch) one that targets non-polar triacylglycerols (TAGs) and more polar phospholipids, it is likely that the FAs detectable in the wine/vinegar products explored here, and also those present in Zhenjiang aromatic vinegars [32], are present as free non-glycerol-esterified species and their corresponding anions, and this would account for their higher levels detectable in these studies than those reported in [43]. Indeed, fermentation processes readily induce the hydrolysis of TAGs to free FAs, together with mono- and diacylglycerol adducts, and free glycerol [44]; such FAs will be expected to contribute towards the food pH values determined here. Similarly, Phan et al. [45] found a broad spectrum of lipidic species, specifically TAGs, polar lipids, free FAs, sterols, and cholesterol esters present in pinot noir wines.

The official AOAC gravimetric method for lipid determination employed in the current study involves an acid hydrolysis step involving HCl in any case, followed by extraction with mixed ethers, i.e., both diethyl and petroleum ethers. Hence, the HCl added will be sufficient to hydrolyze any residual TAGs present to free FAs and glycerol, and also fully protonate the former so that they are extractable as such into ether solvents. Indeed, it has been demonstrated that such free FAs are readily soluble and extractable into these ether solvent systems [46,47]. Hence, the passage of lipidic species from grapes and/or micro-organisms to finalized bottled wine and vinegar products has been confirmed in further investigations.

Interestingly, ^1^H NMR analysis of ^2^H_2_O extracts of the FCLO product investigated found proportionately high concentrations of free FAs and free glycerol therein (data not shown). These FAs were mainly present as PUFAs, as would be expected from the overall lipid composition of this product which contains high levels of omega-3 FAs as TAG species prior to fermentation induction. This observation is fully consistent with the ability of lactobacilli-mediated fermentation processes to partially hydrolyze TAGs in such a product. High levels of the short-chain organic acids propionic and acetic acids (as their propionate and acetate anions in neutral solution media), both lactobacilli fermentation catabolites, the former arising from the metabolic reduction of lactate [48], were also detectable in these extracts. These results will be reported in detail elsewhere.

## 4. Limitations of the Study

One important limitation of this study is the limited sample sizes of some of the fermented food sampling classes incorporated into our primary experimental design. This was largely a consequence of only small numbers of differing fermented food products being available for purchase locally, for example vegetable sauce and fish products. However, it should be noted that the cheese and wine/vinegar classifications had BA contents and patterns which markedly contrasted with those of the other fermented food groups evaluated. These differences, along with those for other food quality markers observed (Table 3, Figure 1, Figure 3, Figure 4 and Figure 5), were found to be very highly statistically significant, even with these limited sample sizes. Hence, this did not present a major constraining issue. Moreover, the performance of additional MV analyses on a revised model including a combined fish, fish sauce/paste, and vegetable sauce classification (on the basis of only a limited level of significant differences between them) with *n* = 17 overall served to overcome this problem (Figure 6), and this incentive did not distract from the main objectives and focus of the investigation in view of their predominant MV similarities in BA contents. However, univariate analysis found that the mean spermidine concentration was significantly higher in fermented vegetable sauces than it was in corresponding fish products (Table 3), and vice-versa for mean 2-phenylethylamine levels (Table 7). Further evidentiary support was provided by data analysis strategies applied, which were highly rigorous, and included the preliminary tracking of sample outliers. Furthermore, rigorous Welch tests were implemented for the ANOVA models employed, and either Bonferroni or FDR corrections were applied for *post*-*hoc* “between-fermented food classification” tests in order to circumvent potential problems with false positives (type I errors).

Another limitation of the current study was the unavailability of differing manufacturing sources of FCLO products, and therefore unlike other fermented food products assessed here, statistical evaluations involved an investigation of 10 separate, randomly-selected batches of a single product, both separately (Table 5) and jointly with all other classes involved in the primary statistical analysis conducted (Table 7, and Figure 5 and Figure 6). However, the very wide between-batch variance of all FCLO samples explored facilitated this approach.

Finally, one further limitation is the poor specificity and interpretability of the TBARS method employed for the quality assessment of fermented food products here, specifically for assessments of their degrees of lipid peroxidation. However, one major precautionary step was taken in this study to minimize problems and potential interferences in this assay system, and this involved the avoidance of an aldehydic artefact-forming heating stage. Future investigations of the lipid oxidation status of fermented foods should therefore employ more reliable and specific methodologies such as those involving high-resolution ^1^H NMR analysis for the direct, simultaneous, multicomponent analysis of a series of both primary and secondary lipid oxidation products, e.g., conjugated hydroperoxydienes and their aldehydic fragmentation products, respectively. This protocol may be applied directly to solution-state products, or indirectly to either aqueous or lipid/deuterochloroform extracts of fermented food products.

## 5. Conclusions

This study demonstrated that almost all fermented foods tested had total BA levels which lay below the maximum recommended values for them. A composite application of univariate and MV chemometrics techniques clearly demonstrated that the MV approach applied was valuable for discriminating between fermented wine/vinegar products and cheeses, and the distinction between these two fermented food classes and a combination of fish, fish sauce/paste, and vegetable sauce products. Further MV analysis performed on CS-normalized BA profiles revealed distinctive patterns for cheese (cadaverine only), FCLOs (the aromatic BAs 2-phenylethylamine, tyramine, and tryptamine), and wine/vinegar products (pathway-associated putrescine, spermidine, and spermine). Such distinctive signatures for fermented food BA contents may offer useful information regarding the nature of, and regulatory conditions employed for, fermentation processes utilized during their commercial production.

The simultaneous untargeted analysis of eight or more BAs using the LC-MS/MS analysis strategy employed here offers major advantages which are unachievable by alternative, more targeted techniques with the ability to determine only single or very small numbers of chemometrically-important analytes. Notably, the diagnostic potential of a series of n (for example, five or more) BA content analyte variables in a MV chemometrics investigation offers major advantages over the analytical acquisition of only a single possible marker. Indeed, food sample patterns of BAs and related food quality indices, which are characteristic of a particular fermented food product classification, will be expected to provide a much higher level of statistical power, reliability, and confidence concerning the accurate distinction between these classifications, and their accurate and selective assignment to one of them, than that discernable from a single BA analyte level only. Secondly, the patterns of BAs and associated food quality criteria determined, together with their correlations to particular factors or components (predominantly linear, but occasionally quadratic or higher combinations of predictor BA and supporting variables), may potentially serve to supply extensive information regarding the sources of such BAs, bacterial, commercial, or otherwise.

## Figures and Tables

**Table 1:** Details of fermented food products investigated for each classification.

Fermented Food Classification	Products Investigated
**Cheeses**	Full-fat pasteurized cow’s milk soft cheese (washed with brandy); full-fat French cow’s milk soft-ripened cheese; semi-soft washed rind Limberger cheese; full-fat pasteurized cow’s milk soft cheese; French cow’s milk soft cheese.
**Fish**	Pickled mud fish; pickled gourami fish; dried gourami fish; salted crab.
**Fish Sauce/Paste**	Loc fish sauce; scad fish sauce; anchovy fish sauce; Vietnamese fish sauce (×2); Thai fish sauce; standard U.S. fish sauce; shrimp paste (×2).
**Vegetable Sauce**	Bean curd; chili bean sauce; kimchi sauce; spicy tofu sauce.
**Wine/Vinegar**	Balsamic vinegar (×2); red wine vinegar; Casella wine.

**Table 2:** Biogenic amines (BA) contents and quality indices of fermented foods investigated. Mean ± SEM BA levels, and titratable acidity (TA), pH, total lipid, thiobarbituric acid-reactive substances (TBARS) and (TBARS):(total lipid) ratio values, for five classes of fermented food products (cheese, fish, fish sauce/paste, vegetable sauce, and wine/vinegar) purchased at a range of U.S. retail outlets (bracketed numbers represent the number of different products analyzed for each classification).

BA Variable/ppm	Cheese (5)	Fish (4)	Fish Sauce (9)	Vegetable Sauce (4)	Wine/Vinegar (4)
Cadaverine	191.6 ± 99.8	30.7 ± 5.6	45.2 ± 8.2	30.6 ± 14.0	0.7 ± 0.7
Histamine	5.7 ± 1.6	10.6 ± 2.9	20.0 ± 5.8	17.7 ± 9.5	1.9 ± 1.9
2-Phenylethylamine	11.1 ± 6.8	13.1 ± 8.0	8.3 ± 4.2	5.00 ± 5.00	nd
Putrescine	21.2 ± 14.9	14.9 ± 7.2	18.9 ± 5.1	18.4 ± 7.7	3.3 ± 0.15
Spermidine	9.6 ± 3.2	10.9 ± 2.5	15.0 ± 1.9	24.5 ± 10.1	4.4 ± 1.5
Spermine	3.5 ± 2.2	12.6 ± 4.5	18.7 ± 3.0	11.4 ± 4.3	1.5 ± 1.5
Tryptamine	7.0 ± 5.8	2.0 ± 0.7	5.6 ± 1.7	3.4 ± 1.0	nd
Tyramine	69.4 ± 42.5	8.9 ± 3.7	17.2 ± 4.0	36.4 ± 20.5	0.6 ± 0.4
Total BAs	322.2 ± 166.0	103.8 ± 12.7	155.9 ± 18.7	147.9 ± 56.2	12.4 ± 5.5
Titratable Acidity (g acid/100 g)	1.3 ± 1.1	0.6 ± 0.2	0.6 ± 0.1	0.7 ± 0.2	3.6 ± 1.2
pH	6.09 ± 1.38	6.33 ± 0.62	5.47 ± 0.28	5.18 ± 0.53	2.99 ± 0.20
Total Lipid (% *w*/*w*)	23.3 ± 2.0	6.9 ± 2.7	4.2 ± 1.4	5.1 ± 2.8	1.1 ± 0.2
TBARS Value (ppm)	0.07 ± 0.05	0.35 ± 0.14	0.47 ± 0.27	0.09 ± 0.03	0.83 ± 0.51
10^2^.(TBARS):(Total Lipid) Ratio (ppm(% *w*/*w*)^−1^)	0.5 ± 0.4	10.6 ± 6.85	33.1 ± 22.3	5.6 ± 3.55	98.3 ± 45.7

nd: not determined.

**Table 3:** Statistical significance and nature of differences between the mean BA contents and other food quality indices for fermented food products. Both robust Welch and Bonferroni-corrected *post*-*hoc* ANOVA test significance (*p*) values are provided. Abbreviations: ns, not statistically significant. * These values were close to statistical significance, but did not attain a *p* value of ≤0.05 with the robust Welch test.

BA/Index	Welch Test (WT) *p* Value	*Post*-*hoc* Significant Differences (All *p* < 0.05: Bonferroni Test)
Cadaverine (ppm)	0.0016	Cheese > Wine/Vinegar; Cheese > Fish Sauce/Paste; Cheese > Fish; Cheese > Vegetable Sauce
Histamine (ppm)	0.087 *	All ns
2-Phenylethylamine (ppm)	ns	All ns
Putrescine (ppm)	0.068 *	All ns
Spermidine (ppm)	0.029	Vegetable Sauce > Wine/Vinegar; Vegetable Sauce > Cheese; Vegetable Sauce > Fish
Spermine (ppm)	0.010	Fish Sauce/Paste > Wine/Vinegar; Fish Sauce/Paste > Cheese
Tryptamine (ppm)	ns	All ns
Tyramine (ppm)	0.021	Cheese > Wine/Vinegar
Total BAs	2.84 × 10^−4^	Cheese >> Wine/Vinegar
Titratable acidity (g acid/100 g)	0.024	Wine/Vinegar > Fish; Wine/Vinegar > Fish Sauce/Paste; Wine/Vinegar > Vegetable Sauce; Wine/Vinegar > Cheese
pH	6.91 × 10^−4^	Wine/Vinegar < Fish; Wine/Vinegar < Fish Sauce/Paste; Wine/Vinegar < Vegetable Sauce Wine/Vinegar < Cheese
Total lipid (% *w*/*w*)	1.09 × 10^−3^	Cheese > Wine/Vinegar; Cheese > Fish Sauce/Paste; Cheese > Fish; Cheese > Vegetable Sauce
TBARS value (ppm)	ns	ns
10^2^.(TBARS):(Total Lipid) ratio (ppm(% *w*/*w*)^−1^)	ns	Wine/Vinegar > Fish; Wine/Vinegar > Vegetable Sauce; Wine/Vinegar > Cheese

ns: not statistically significant.

**Table 4:** PCA loadings vectors for BAs and additional fermented food analyte parameters (including total lipid contents, and pH and TA values) for a 5 PC-limited model performed with varimax rotation and Kaiser normalization. Percentage variance contributions for PCs 1–5 and their (unrotated) analysis eigenvalues are also listed. Bold numbers are for a purpose specified in the Figure legends.

PC (Unrotated Eigenvalue):	PC1 (4.58)	PC2 (2.26)	PC3 (1.78)	PC4 (1.28)	PC5 (0.89)
**% Variance Contribution**	26.6	16.9	12.2	16.1	11.2
2-Phenylethylamine	**0.71**	−0.23	**0.43**	0.03	−0.06
Cadaverine	**0.90**	−0.07	−0.19	0.21	−0.04
Histamine	0.25	**0.45**	**0.63**	0.025	0.32
Putrescine	**0.57**	−0.04	−0.19	**0.42**	**0.50**
Spermidine	0.14	−0.19	0.20	0.06	**0.78**
Spermine	−0.08	−0.06	**0.85**	0.22	0.12
Tryptamine	**0.79**	0.07	0.11	0.13	0.30
Tyramine	**0.88**	−0.11	0.03	0.15	−0.03
Titratable acidity (TA)	−0.11	0.17	−0.30	**−0.86**	−0.09
pH	0.29	−0.10	0.04	**0.92**	−0.07
TBARS value	−0.08	**0.95**	0.08	−0.05	−0.05
Total lipid	**0.45**	−0.31	−0.21	0.37	**−0.61**
(TBARS):(Lipid) ratio	−0.14	**0.92**	−0.07	−0.23	−0.08

**Table 5:** (BA concentrations (ppm) of *n* = 10 separate batches of a FCLO product. Total BA and corresponding mean ± SEM values are also provided. Histamine and spermine were undetectable in all samples analyzed.

	**FCLO Batch**										
**Biogenic Amine (ppm)**	**1**	**2**	**3**	**4**	**5**	**6**	**7**	**8**	**9**	**10**	**Mean ± SEM**
2-PE	86	103	50	17	76	0	1.4	0	1.9	1.3	33.7 ± 13.0
Tyramine	70	88	43	8	32	0	1.8	0	1.1	0	24.4 ± 10.3
Tryptamine	35	24	26	3	8	0	0	0	1.7	1.5	9.9 ± 4.2
Cadaverine	23	11	25	0	7	0	0	0	0	0	6.6 ± 3.1
Putrescine	14	10	14	0	0	0	0	0	0	0	3.8 ± 2.0
Spermidine	0	4	0	0	0	0	0	0	0	0	0.4 ± 0.4
Total	228	240	158	28	123	0	3.2	0	4.7	2.8	78.8 ± 31.3

**Table 6:** PCA loadings vectors for FCLO BAs in a two PC-limited PCA model performed with varimax rotation and Kaiser normalization. Percentage variance contributions for these PCs and their (unrotated) analysis eigenvalues are also listed. Bold numbers are for a purpose specified in the Figure legends.

PC (unrotated Eigenvalue)	PC1 (4.03)	PC2 (1.58)
**% Variance Contribution**	52.8	40.6
2-PE	0.35	**0.84**
Tyramine	**0.54**	**0.84**
Tryptamine	**0.93**	0.33
Cadaverine	**0.99**	−0.01
Putrescine	**0.95**	0.23
Spermidine	−0.11	**0.93**

**Table 7:** Univariate statistical significance and nature of differences observed between the mean CS-normalized, glog-transformed, and autoscaled BA contents of fermented food samples (cheese, FCLO, fish, fish sauce/paste, vegetable sauce, and wine/vinegar products) in a completely randomized, one-way ANOVA model. The significance of FDR-corrected *post*-*hoc* ANOVA tests are also provided (significant differences are ranked in order of their decreasing statistical significance, i.e., increasing *p* value). The “between-fermented food class” source of variation was not statistically significant for putrescine, spermidine, spermine, histamine, or tyramine when tested in this model.

BA	FDR-Corrected *p* Value	Significant post-hoc *ANOVA* Differences
Cadaverine	1.49 × 10^−5^	Cheese > FCLO; Cheese > Vegetable Sauce; Cheese > Wine/Vinegar; Fish > FCLO; Fish Sauce > FCLO; Vegetable Sauce > FCLO; Fish > Wine/Vinegar; Fish Sauce > Wine/Vinegar; Vegetable Sauce > Wine/Vinegar.
2-Phenylethylamine	8.25 × 10^−4^	FCLO > Cheese; FCLO > Fish; FCLO > Fish Sauce; FCLO > Vegetable Sauce; FCLO > Wine/Vinegar; Fish > Vegetable Sauce.
Tryptamine	2.93 × 10^−2^	FCLO > Cheese; FCLO > Fish; FCLO > Fish Sauce; FCLO > Vegetable Sauce; FCLO > Wine/Vinegar.

## Supplementary Materials

The following are available online at https://www.mdpi.com/2304-8158/9/12/1807/s1, S1: Summary of Antioxidant Activities of BAs, S2: Potential Adverse Health Effects of Dietary Bas, S3: Outline of Analytical Techniques Available for BA Determinations and the Screening of BA-Generating Bacteria in Foods.
