# Supplementary material for: Extensive Chemometric Investigations of Distinctive Patterns and Levels of Biogenic Amines in Fermented Foods: Human Health Implications
Source: Foods. 2020 Dec 5;9(12):1807. doi: 10.3390/foods9121807 (PMC7762153; doi:10.3390/foods9121807)
Supplement: Supplementary file 1 [file foods-09-01807-s001.pdf]

SUPPLEMENTARY MATERIALS

S1 Summary of Antioxidant Activities of BAs

Both the polyamines spermine and spermidine, at added concentrations of 0.49-2.97 and 0.69-4.13 mmol./kg respectively, offered powerful antioxidant activities against PUFA peroxidation in soybean oil [S1] (this investigation used the Rancimat® methodology which involved heating the oils to 110°C). Intriguingly, both these BAs were demonstrated to be more effective antioxidants than butylated hydroxyanisole, butylated hydroxytoluene, α-tocopherol, and rosemary and green tea extracts. Several mechanisms of action have been proposed for the antioxidant capacities of BAs, and these are highly molecularly-specific. For example, tyramines demonstrate peroxyl radical-scavenging activities, as reported by Stevenato *et al.* [S2], who utilised an *in vitro* investigation. However, the antioxidant characteristics of the aliphatic polyamines spermine, spermidine, putrescine, cadaverine, histamine and tryptamine were attributable to the direct neutralisation of O_2-consuming species, presumably carbon-centred pentadienyl radicals, which represent the first primary lipid-based radical species formed during the lipid peroxidation process [S2]. Indeed, such polyamines have been shown to exert powerful scavenging activity towards such PUFA-sourced alkyl radicals [S3]. Regardless of the mechanisms involved, all amines tested successfully blocked O_2 consumption by peroxidising linoleoylglycerols in an appropriate model system [S2].

S2 Potential Adverse Health Effects of Dietary BAs

Irrespective of their clinical benefits, the therapeutic employment of monoamine oxidase inhibitor (MAOI) drugs has been somewhat limited in view of their possible interactions with the consumption foods containing high levels of tyramine [17]. Indeed, the 'MAOI diet' restricts the intake of dietary tyramine, a BA which is largely present in fermented food sources such as notably aged cheese, cured meats, sauerkraut, tap beer and soy sauce [61]. The carcinogenic potential of nitrosamines arising from the reactions of secondary BAs, e.g. cadaverine and putrescine, with nitrite, are also worthy of consideration [S4]. Additionally, BAs have been implicated as causative agents responsible for selected food poisoning episodes, particularly those induced by excessive histamine ingestion. Histamine fish poisoning (otherwise known as scombroid poisoning) is caused by the dietary consumption of elevated levels of histamine-rich fish products (e.g., tuna, swordfish, marlin, sardines and mackerel). Indeed, these excessive concentrations of histamine are generated in fish from the enzymatic transformation of a histidine substrate by histidine carboxylase, which arises from a bacterial source. Symptoms usually appear very rapidly following fish consumption (generally within several hr.), and these encompass a tingling of the mouth and lips, a 'peppery' taste sensation, headaches, dizziness, a skin rash and consequent skin itching. Although the usual longevity of such symptoms is 4-6 hr., those afflicted may be treated with antihistamines. Notwithstanding, toxicological effects induced by the ingestion of BAs in other food sources such as red wine and cheese are only occasionally reported. Despite this, the ingestion of high levels of tyramine in cheese products is most notable when experienced by subjects receiving MAOI antidepressant therapies (this is otherwise known as the 'cheese reaction') [S5]. To date, there appears to be only limited specific legislation available regarding BA food contents. However, there are specific limits for the histidine content of fish products

(Commission Regulation (EC) 2073/2005), but only recommendations or suggestions for the upper threshold limits are available for the BA contents of alternative foods. Examples of these limits are 100 mg histamine/kg (100 ppm) of foods [22], and 2 mg histamine/L (2 ppm) for alcoholic beverages. Toxic dose levels for histamine and tyramine in alcoholic beverages are considered to be 8-20 mg/L and 25-40 mg/L respectively; however, only 3 mg/L of 2-phenylethylamine is indicated to cause deleterious health effects [S6].

Along with the potential adverse effects exerted by the human ingestion of excessive levels of histamine, tyramine is a vasoactive amine that enhances hypertension, a process inducing pain. This BA can also give rise to cerebral vasoconstriction, and subsequent rebound vasodilation, which induces migraine attacks in susceptible individuals. Such symptoms usually appear 1-12 hr. following the ingestion of tyramine-rich foods, and a dose of only 10 mg tyramine has been linked to migraine onset [S7]. However, ingested amounts of only 6 mg may cause migraine problems in patients undergoing therapy with MAO inhibitors. Alcohol ingestion has been shown to facilitate the *in vivo* absorption of tyramine. Aged or fermented foods and beverages are rich in tyramine, and these include fish such as pickled herring, peanuts, tinned figs, dried meat products, together with a wide variety of cheese, sausage, wine, beer and vinegar commodities [S7].

Tyramine is predominantly produced in fermented foods via the actions of bacterial tyrosine decarboxylase, and serves as one of the most predominant BAs available in such products. A range of lactic acid bacteria, e.g. lactobacilli, carnobacteria and enterococci, which are all active during the generation of most fermented foods, are equipped with tyrosine decarboxylation enzymes for tyramine formation, although this process displays critical dependencies on species, strains and conditions [S8-S11]. Besides tyramine, excessive intakes of 2-phenylethylamine can also give rise to the onset of migraines in humans [S12]. Although its sources are described as diverse, this BA, which is present at levels of *ca.* 100 ppm in cheese and chocolate [3], has also been demonstrated to be formed during food fermentation episodes [S13], and it has been suggested that it may be generated from the actions of tyrosine-decarboxylating lactobacilli enzymes on phenylalanine [S12]. However, the possible prior metabolic transformation of this amino acid to tyrosine by phenylalanine hydroxylase noted above [40] may also serve as a mechanism for tyramine production.

Moreover, both putrescine and cadaverine have displayed *in vitro* cytotoxic actions towards human intestinal cell cultures in a real-time cell analysis (RTCA) system [S13]. Indeed, the mode of cytotoxic action for both these BAs involved cell necrosis, although neither gave rise to apoptosis. Selected lactobacilli strains readily degrade LL-arginine [S14-S16] to putrescine, this amino acid representing one of the most concentrated amino acids present in fruit juices and wines. Furthermore, in Argentinian red and white wines, the protease formed from *Oenococcus oeni* has the ability to degrade nitrogenous macromolecules therein; in a quantitative context, putrescine-generating arginine has been found to be one of the most portentous amino acids liberated from such protease activity [S17]. Moreover, Halaasz *et al.* (1994) [4] found a positive relationship between putrescine levels and those of microflora present in vegetables.

S3 Outline of Analytical Techniques Available for BA Determinations and the Screening of BA-Generating Bacteria in Foods

Previously, methods available for the determination of biogenic amines in foods have included relatively simple approaches based on paper chromatography or spectrofluorimetry, and also more complex analytical strategies, including the automated detection of microbial metabolic actions, and automated conductance monitoring [S18,S19]. Along with the determination of BA catabolites themselves, the early detection and identification of BA-generating bacterial sources is also of much importance. Indeed, BA-forming micro-organisms may be identified via the use of differential culture media supplemented with a colourimetric pH indicator to monitor differential strains [S20], but these techniques may be limited by the inability of lactic acid bacteria to produce BAs following prolonged periods of storage, or the use of synthetic media for the cultivation of isolated strains [S21]. However, currently the detection and quantification of BAs is largely achieved by LC and gas chromatographic (GC), and capillary electrophoresis (CE) methods. LC-based detection techniques usually involve spectrophotometry (including diode array detection in the UV region), fluorescence and MS attachments [S22-S28]. Furthermore, reliable sensor devices, enzymatic and immunoassay methods, in addition to commercially-available testing kits available for the expedient determination of tyramine and histamine in wine products, are available for application [S29].

Nevertheless, the employment of rapid and reliable probes, which are usually based on PCR biotechnologies with selected target genes, has permitted the accurate screening of BA-generating bacteria in a range of fermented beverages [S30-S32]. Indeed, a series of sophisticated multiplex and real-time quantitative PCR techniques have been developed for the identification of lactic acid bacteria in fermented foods, for example during cheese manufacture, and wine and cider fermentation processes [S32-S35].

Supplementary Materials References

[S1] Mendonca, A. C. Atividade an antioxidante de poliaminas e comparacao com produtos naturais e sinteticos. PhD Thesis **2009**, Faculdade de Farmácia da University de Federal de Minas Gerais (UFMG), Belo Horizonte, MG, Brasil.

[S2] Stevanato, R.; Mariangela, B.; Fabris, S. In vitro preliminary evidences on the antioxidant properties of biogenic amines. *Pharmacol. Pharm.* **2013**, *4*, 696-700. http://www.scirp.org/journal/pp) http://dx.doi.org/10.4236/pp.2013.49097

[S3] Fujisawa, S.; Kadoma, Y. Kinetic evaluation of polyamines as radical scavengers. *Anticancer Res*. **2005**, *25*(2A), 965-970.

[S4] Ten Brink, B.; Damink, C.; Joosten, H. M. L. J,; Huisint-Veld, J. H. J. Occurrence and formation of biologically amines in food. *Int. J. Food Microbiol.* **1990**, *11*, 73–84.

[S5] Silla Santos, M. H. Biogenic amines: their importance in foods. *Int. J. Food Microbiol.* **1996**, *29*, 213–231.

[S6] Soufleros, E.; Barrios, M. L.; Bertrand, A. Correlation between the content of biogenic amines and other wine compounds. *Am. J. Enol. Viticul.* **1998**, *49*, 266–269.

[S7] Costa, M. R.; Glória, M. B. A. Migraine and diet. Editor(s): Caballero, B. *Encyclopedia of Food Sciences and Nutrition* (Second Edition), Cambridge, MA, USA, Academic Press, **2003**, 3940-3947. ISBN 9780122270550, https://doi.org/10.1016/B0-12-227055-X/00783-5.

[S8] Gardini, F.; Martuscelli, M.; Caruso, M. C.; Galgano, F.; Crudele, M. A.; Favati, F.; Guerzoni, M. E.; Suzzi, G. Effects of pH, temperature and ClNa concentration on the growth kinetics, proteolytic activity and biogenic amine production of Enterococcus faecalis. *Int. J. Food Microbiol.* **2001**, *64*, 105-117.

[S9] Gardini. F.; Bover-Cid, S.; Tofalo, R.; Belletti, N.; Gatto, V.; Suzzi, G.; Torriani, S. Modeling the aminogenic potential of Enterococcus faecalis EF37 in dry fermented sausages through chemical and molecular approaches. *Appl. Environ. Microbiol.* **2008**, *74*, 2740-2750.

[S10] Marcobal, A.; Martín-Alvarez, P. J.; Moreno-Arribas, M. V.; Muñoz, R. A multifactorial design for studying factors influencing growth and tyramine production of the lactic acid bacteria Lactobacillus brevis CECT 4669 and Enterococcus faecium BIFI-58. *Res. Microbiol.* **2006**, *157*, 417-424.

[S11] Coton, E.; Coton, M. Evidence of horizontal transfer as origin to strain variation of the tyramine production trait in Lactobacillus brevis. *Food Microbiol.* **2009**, *26*, 52-57.

[S12] Millichap, J. G.; Yee, M. M. The diet factor in pediatric and adolescent migraine. *Pediatr. Neurol.* **2003**, *28*, 9-15.

[S13] Del Rio, B.; Redruello, B.; Linares, D. M.; Ladero, V.; Ruas-Madiedo, P.; Fernandez, M.; Martin, M. C.; Alvarez, M. A. The biogenic amines putrescine and cadaverine show in vitro cytotoxicity at concentrations that can be found in foods. *Sci. Rep.* **2019**, *9*(1), 120. https://doi.org/10.1038/s41598-018-36239-w

[S14] Manca de Nadra, M.C.; Pesce de Ruiz Holgado, A.; Oliver, G. Utilization of LL-arginine by Lactobacillus buchneri: arginine deiminase. *Milchwissenchaft* **1981**, *36*, 356-359.

[S15] Manca de Nadra, M.C.; Nadra Chaud, C.; Pesce de Ruiz Holgado, A.; Oliver, G. Arginine metabolism in Lactobacillus leichnannii. Current Microbiology **1986**, *13*, 155-158.

[S16] Manca de Nadra, M. C.; Pesce de Ruiz Holgado, A.; Oliver, G. Arginine dihydrolase pathway in Lactobacillus buchneri:a review. *Biochimie* **1988**, *70*, 367-374.

[S17] Manca de Nadra, M. C.; Farõ Â as, M.E.; Moreno-Arribas, M.V.; Pueyo, E.; Polo, M.C. A proteolytic effect of Oenococcus oeni on the nitrogenous macromolecular fraction on red wine. *FEMS Microbiol. Lett.* **1999**, *174*, 41-47.

[S18] Onal, A. A review: current analytical methods for the determination of biogenic amines in food. *Food Chem.* **2007**, *103*, 1475–1486.

[S19] Spano, G.; Russo, P.; Lonvaud-Funel, A.; Lucas, P.; Alexandre, H.; Grandvalet, C,; Coton, E.; Coton, M.; Barnavon, L,; Bach, B. *et al.* Biogenic amines in fermented foods. *Eur. J. Clin. Nutrition* **2010**, *64*, S95–S100.

[S20] Bover-Cid, S.; Holzapfel, W. H. Improved screening procedure for biogenic amine production by lactic acid bacteria. *Int. J. Food Microbiol.* **1999**, *53*, 33–41.

[S21] Lucas, P. M.; Wolken, W. A. M.; Claisse, O.; Lolkema, J. S.; Lonvaud-Funel, A. Histamine-producing pathway encoded on an unstable plasmid in Lactobacillus hilgardii 0006. *Appl. Environ. Microbiol.* **2005**, *71*, 1417–1424.

[S22] Almeida, C.; Fernandes, J. O.; Cunha, S. C. A novel dispersive liquid–liquid microextraction (DLLME) gas chromatography-mass spectrometry (GC–MS) method for the determination of eighteen biogenic amines in beer. *Food Control* **2012**, *25*(1), 380-388.

[S23] Arce, L.; Ríos, A.; Valcárcel, M. Direct determination of biogenic amines in wine by integrating continuous flow clean-up and capillary electrophoresis with indirect UV detection. *J. Chromatogr. A* **1998**, *803*(1-2), 249-260.

[S24] Cunha, S. C.; Faria, M.A.; Fernandes, J. O. Gas chromatography–mass spectrometry assessment of amines in port wine and grape juice after fast chloroformate extraction/derivatization. *J. Agric. Food Chem.* **2011**, *59*(16), 8742-8753.

[S25] Daniel, D.; dos Santos, V. B.; Vidal, D. T. R., do Lago, C.L. Determination of biogenic amines in beer and wine by capillary electrophoresis–tandem mass spectrometry. *J. Chromatogr. A* **2015**, *1416*, 121-128.

[S26] Gómez-Alonso, S.; Hermosín-Gutiérrez, I.; García-Romero, E. Simultaneous HPLC analysis of biogenic amines, amino acids, and ammonium ion as amino enone derivatives in wine and beer samples. *J. Agric. Food Chem.* **2007**, *55*(3): 608-613.

[S27] Mafra, I.; Herbert, P.; Santos, L.; Barros, P.; Alves, A. Evaluation of biogenic amines in some Portuguese quality wines by HPLC fluorescence detection of OPA derivatives. *Am. J. Enol. Vitic.* **1999**, *50*(1): 128-132.

[S28] Pereira, V.; Pontes, M.; Câmara, J.S.; Marques, J. C. Simultaneous analysis of free amino

acids and biogenic amines in honey and wine samples using in loop ortho-phthalaldeyde derivatization procedure. *J. Chromatogr. A* 2008, *1189*(1–2), 435-443.
[S29] Simon-Sarkadi, L.; Gelencsér, É.; Vida, A. Immunoassay method for detection of histamine in foods. *Acta Aliment Hung.* **2003**, *32*(1), 89-93.

[S30] Marcobal, A.; de las Rivas, B.; Munoz, R. Methods for the detection of bacteria producing biogenic amines on foods: a survey. *J. Con. Prot. Food Safety* **2006**, *1*, 187-196.

[S31] Ladero, V. M.; Linares, D. M.; Fernandez, M.; Alvarez, M. A. Real time quantitative PCR detection of histamine-producing lactic acid bacteria in cheese: relation with histamine content. *Food Res. Int.* **2008**, *41*, 1015-1019.

[S32] Nannelli, F.; Claisse, O.; Gindreau, E.; de Revel, G.; Lonvaud-Funel, A.; Lucas, P. M. Determination of lactic acid bacteria producing BAs in wine by quantitative PCR methods. *Lett. Appl. Microbiol.* **2008**, *47*, 594-599.

[S33] Marcobal, A.; de las Rivas, B.; Moreno-Arribas, M. V.; Munoz, R. Multiplex PCR method for the simultaneous detection of histamine-, tyramine-, and putrescine-producing lactic acid bacteria in foods. *J. Food Prot.* **2005**, *68*, 874-878.

[S34] Lucas, P. M.; Claisse, O.; Lonvaud-Funel, A. High frequency of histamine producing bacteria in the enological environment and instability of the histidine decarboxylase production phenotype. *Appl. Environ. Microbiol.* **2008**, *74*, 811-817.

[S35] Wongsariya, K.; Bunyapraphatsara, N.; Yasawong, M.; Chomnawang, M. T. Development of molecular approach based on PCR assay for detection of histamine producing bacteria. *J. Food Sci, Technol.* **2016**, *53*(1), 640-648. doi:10.1007/s13197-015-1982-1
